# Labour-type physical activity, alcohol use and hypertension in rural older adults in Northeast China

**DOI:** 10.3389/fpubh.2026.1748721

**Published:** 2026-02-25

**Authors:** Yongheng Zhao, Gaixia Hou, Yimeng Gu, Zhuangzhuang Guo, Dehui Zhang, Xuefeng Xi, Limeng Liu, Lizhen Ning

**Affiliations:** 1 School of Wushu, Henan University, Kaifeng, China; 2 Institute of Gerontology, Henan University, Kaifeng, China; 3 Graduate School, Kyungil University, Gyeongsan-si, Republic of Korea; 4 Dongcheng Community Health Service Center, Wangkui County, Heilongjiang, China; 5 School of Physical Education and Health Sciences, Mudanjiang Normal University, Mudanjiang, China; 6 School of Physical Education, Suihua University, Suihua, China

**Keywords:** alcohol consumption, cold-climate environment, hypertension, occupational physical activity, population-attributable risk, rural older adults

## Abstract

**Background:**

Hypertension is highly prevalent in older adults, yet evidence from resource-limited rural settings remains limited. In Northeast China, older residents are chronically exposed to cold-climate stress, labour-intensive agricultural routines, and entrenched social drinking norms, which may shape blood pressure risk profiles differently from urban cohorts.

**Methods:**

We analysed data from the 2025 Rural Elderly Health Examination Programme in Wangkui County, Heilongjiang, using a community-based cross-sectional design. Participants aged ≥65 years (*N* = 2,270) completed standardised examinations including bilateral blood pressure measurement, anthropometrics, and questionnaires assessing workload-related physical activity frequency—dominated by farming and domestic labour in this setting (hereafter termed occupational/labour-type physical activity, OPA; sessions/week)—and alcohol drinking frequency (occasions/week). Hypertension was defined as higher-arm SBP ≥ 140 mmHg and/or DBP ≥ 90 mmHg. Associations were estimated using multivariable logistic regression with HC3 robust standard errors, adjusting for age, sex, body mass index, haemoglobin concentration, and winsorized resting heart rate (complete-case *N* = 2,194).

**Results:**

Higher OPA frequency and alcohol drinking frequency were independently associated with greater odds of hypertension. Each additional OPA session per week was associated with a 23% higher odds of hypertension (adjusted OR [aOR] = 1.23, 95% CI: 1.16–1.32), and each additional drinking occasion per week was associated with a 20% higher odds (aOR = 1.20, 95% CI: 1.04–1.40). Estimated population-attributable fractions suggested a substantial potential burden associated with high-frequency OPA (≥3 sessions/week; 34.8%) and a smaller burden associated with any weekly drinking (>0/week; 4.7%); these estimates were interpreted cautiously given the cross-sectional design and the use of odds ratios for a common outcome. Sensitivity analyses using alternative hypertension definitions and continuous SBP/DBP models yielded directionally consistent findings, with steeper OPA gradients at older ages.

**Conclusion:**

In this rural older-adult cohort, workload-related physical activity—reflecting largely non-volitional labour rather than leisure-time exercise—and alcohol use were associated with higher hypertension likelihood. Prevention strategies in cold-climate rural communities may benefit from workload-modification and recovery-protection approaches, safer organisation of labour tasks, and targeted reduction of weekly alcohol use.

## 1 Introduction

As the global population ages, hypertension has become a major public health challenge and a leading contributor to cardiovascular morbidity and mortality in older adults (1, 2). Although the epidemiology of hypertension has been well described in urban and clinic-based cohorts, evidence from rural ageing populations remains comparatively limited, despite their distinct exposure profiles and constrained access to prevention and care (3). Rural older adults in Northeast China, including Wangkui County in Heilongjiang Province, experience a convergence of cold-climate stress, labour-intensive daily routines, and well-established social norms around alcohol use, all of which may influence blood pressure regulation through pathways that differ from those observed in urban settings (4–6).

Physical activity is generally considered protective against hypertension; however, this premise largely derives from studies of voluntary, leisure-time activity performed at moderate intensity with adequate recovery (7–9). In contrast, physical activity in rural agricultural settings often reflects non-volitional, workload-driven occupational or domestic labour. The “physical activity paradox” describes the observation that high volumes of occupational (labour-type) physical activity may fail to confer cardiovascular benefit—and may even be harmful—when compared with leisure-time activity, particularly when work is prolonged, repetitive, and performed with limited autonomy and insufficient recovery (7–10). These characteristics may elicit chronic haemodynamic strain and sustained sympathetic activation, thereby increasing hypertension risk (9, 11). Such effects may be amplified in cold climates, where peripheral vasoconstriction and thermoregulatory demands increase vascular load and neurohumoral activation, intensifying blood pressure responses for a given workload (9, 11).

Alcohol consumption is another salient behavioural exposure in rural Northeast China. Drinking—often involving high-proof Chinese baijiu (a traditional distilled grain spirit, typically 35–60% alcohol by volume [ABV], commonly distilled from fermented sorghum)—remains embedded in social interaction and community norms (12, 13). While alcohol is a recognised risk factor for hypertension, its impact may be underestimated when examined without considering the sociocultural and environmental context in which drinking occurs (6, 14). In rural settings, alcohol use may co-occur with heavy labour and cold exposure, creating a compound exposure profile that plausibly increases cumulative vascular burden beyond either behaviour alone (6, 14).

Against this background, two critical gaps remain. First, most hypertension-prevention paradigms implicitly treat “physical activity” as uniformly health-promoting, which may not apply in populations where activity is predominantly labour-driven and non-volitional. Second, the joint influence of OPA and alcohol drinking in cold-climate rural ageing environments has not been sufficiently characterised using measurement-based hypertension definitions together with sensitivity analyses using alternative outcome definitions and model specifications. Clarifying these context-dependent relationships is essential to avoid misdirected public health messaging—such as indiscriminately promoting “more physical activity”—in settings where workload reduction and recovery protection may be more relevant.

Therefore, this study examined the associations of OPA frequency and alcohol drinking frequency with measurement-based hypertension among community-dwelling older adults in rural Northeast China. We posited that higher frequencies of OPA—reflecting chronic occupational workload rather than structured exercise—would be associated with increased odds of hypertension, consistent with the physical activity paradox. We also evaluated whether alcohol drinking frequency was associated with hypertension risk and explored combined exposure patterns and effect heterogeneity to inform context-sensitive prevention strategies. By reframing physical activity as a structurally constrained exposure rather than a purely discretionary health behaviour, our findings aim to support more appropriate hypertension prevention in rural ageing populations—emphasising workload modulation (e.g., mechanisation, task redistribution, rest scheduling), alcohol-risk reduction, and cold-environment risk management, rather than generic recommendations to increase activity volume (15–17). Ultimately, this work contributes to a more nuanced understanding of cardiovascular risk in rural communities and provides evidence to guide culturally and environmentally tailored policy responses for healthy ageing (18, 19).

## 2 Methods

### 2.1 Study design and setting

This study employed a community-based, cross-sectional observational design and is reported in accordance with the Strengthening the Reporting of Observational Studies in Epidemiology (STROBE) statement (20). The research was conducted in Wangkui County, Suihua City, Heilongjiang Province, a representative cold-climate agricultural region in Northeast China. The area is characterised by a high reliance on manual labour, prolonged winter seasons, and limited access to specialised healthcare services—structural conditions known to influence cardiovascular risk among older adults (21, 22). Wangkui County reflects the core demographic and agricultural characteristics of the Songnen Plain, making it an informative micro-context for examining workload-related vascular strain in Northeast China.

To contextualise the cold-climate setting, climatological normals for Wangkui (1991–2020) indicate that the cold season is prolonged and severe. Mid-winter temperatures are notably low, with a January daily mean of approximately −19.6 °C, a mean daily minimum of −24.4 °C, and record lows reaching −39.1 °C (23). Although individual-level cold exposure was not directly measured in this database, these climatological data provide an interpretable environmental background against which labour-type activity and alcohol-related vascular strain may operate.

All examinations were organised by the Wangkui County Health Bureau and conducted by the Wangkui County Centre for Disease Control and Prevention (CDC) as part of the 2025 Rural Elderly Health Examination Programme, a routine public health service implemented under national health policy frameworks (24). Clinical assessments, fasting blood sampling, and anthropometric measurements were performed by uniformly trained physicians and nurses following standardised national procedures (25). Laboratory analyses were conducted at the Wangkui County Central Clinical Laboratory, utilising provincially accredited quality-control processes (26). The programme operates on an annual (calendar-year) service cycle. In Wangkui County, examinations are centrally delivered at community health centres (community hospitals), and eligible residents attend voluntarily within the annual examination window following standardised, stepwise community notification. As part of routine public health service delivery, examinations are implemented throughout the year, including winter months, and are not administratively designated to pause during peak agricultural periods (e.g., harvest season). However, because this study used a fully anonymised secondary research database, the dataset retained only the examination year (2025) and did not retain individual-level examination dates (month/season). Consequently, we were unable to conduct season-stratified analyses or directly quantify seasonal influences (e.g., harvest-related workload shifts or extreme winter cold) on OPA reporting. These implementation details were communicated by the Wangkui County health authorities during data governance and authorisation for this secondary analysis.

In November 2025, the Wangkui County Health Bureau granted formal authorisation for Henan University to conduct a secondary scientific analysis of the fully anonymised database. The dataset, originally collected for routine public health service delivery, was subsequently repurposed for secondary epidemiological analysis. All study procedures complied with the Declaration of Helsinki (2013 revision) and relevant provincial ethical regulations (27).

**Figure 1:**
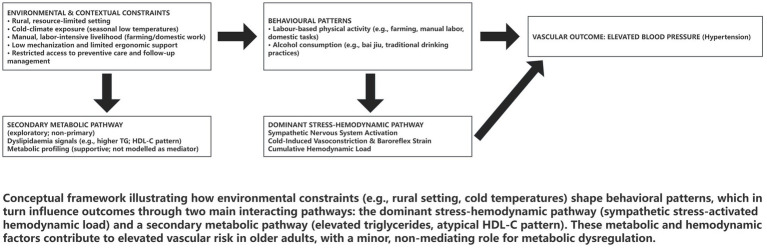
Conceptual framework: hypothesised pathways linking environmental constraints and behavioural stressors (labour-type physical activity and alcohol consumption) to hypertension via stress–haemodynamic and metabolic routes.

**Figure 2:**
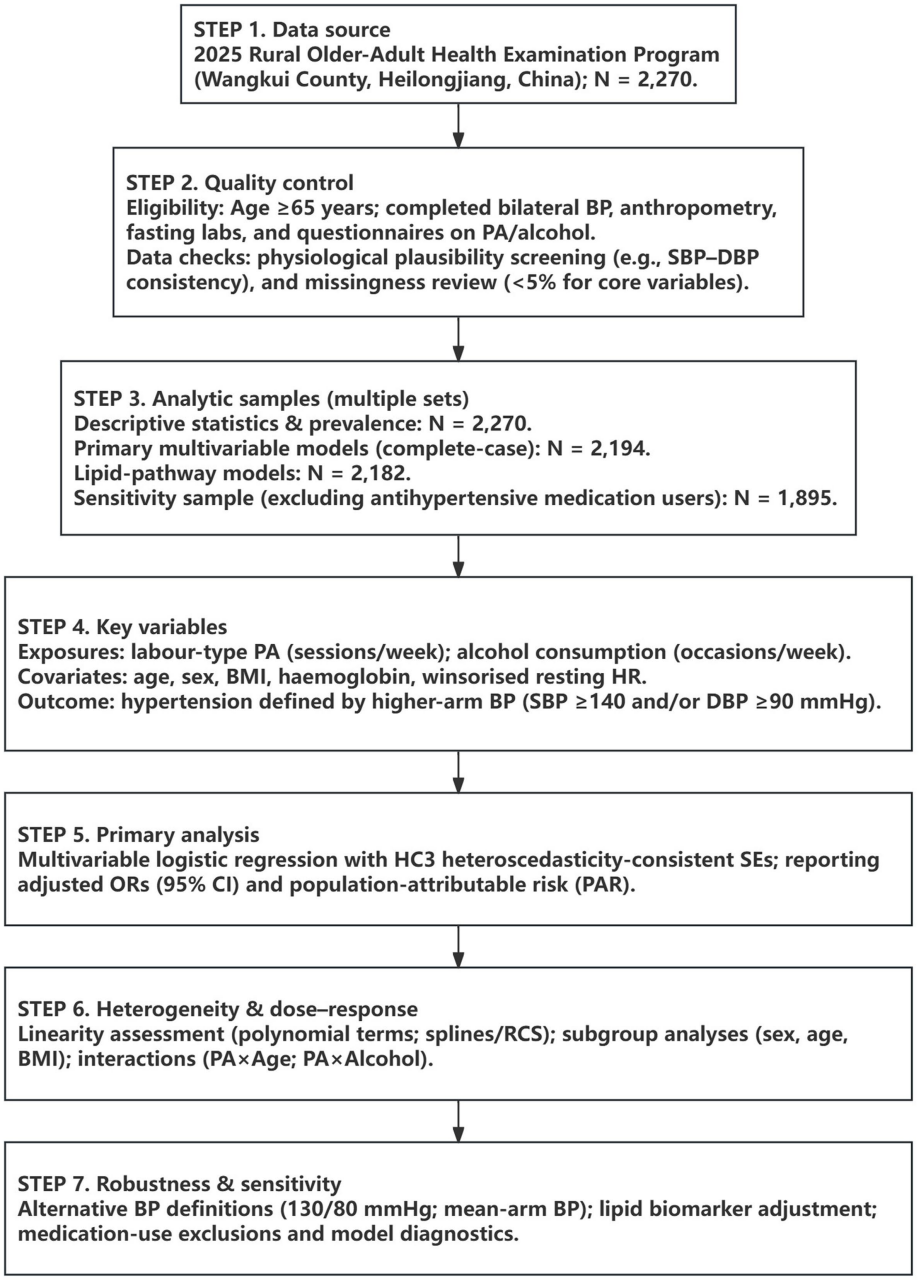
Study workflow: comprehensive analytic pipeline from initial sample selection and validation to primary multivariable models, interaction testing, and robustness validation (*N* = 2,270).

Based on established physiological literature and the contextual constraints of rural Northeast China, we developed a conceptual framework to guide hypothesis testing. As shown in Figure 1, this framework summarises hypothesised pathways linking environmental constraints and behavioural exposures—specifically occupational (labour-type) physical activity (OPA) and alcohol use—to elevated blood pressure via haemodynamic stress and metabolic mechanisms. Figure 2 provides an overview of the study workflow, from initial data collection and sample validation to the final analytic pipeline.

### 2.2 Participants and sampling

The target population comprised all community-dwelling adults aged ≥65 years residing in Wangkui County who were eligible for the 2025 government-organised Rural Elderly Health Examination Programme (28). A census-style recruitment strategy was adopted, whereby village doctors and community health workers utilised official village rosters to notify all eligible residents. Notification followed standardised, stepwise community outreach, and participation was voluntary within the annual examination window. Participants voluntarily attended community health centres (community hospitals) for comprehensive examinations coordinated by the Wangkui County Health Bureau and Centre for Disease Control and Prevention (CDC) (29). A total of 2,270 older adults completed the core examination components, including bilateral systolic and diastolic blood pressure measurements, anthropometric assessments (height, weight, and body mass index [BMI]), fasting venous blood sampling for routine biochemical tests, and standardised questionnaires on occupational (labour-type) physical activity (OPA) and alcohol use. Because the anonymised secondary dataset did not retain month- or season-specific examination dates, individual participation timing could not be characterised.

Physiological plausibility and internal consistency checks were performed in accordance with national technical specifications for older adults health examinations (30). These procedures involved verifying expected measurement relationships (e.g., systolic blood pressure exceeding diastolic blood pressure) and screening values against clinically plausible ranges for blood pressure and lipid profiles. Missingness for core analytic variables (blood pressure, OPA, alcohol use, age, sex, BMI, haemoglobin, and resting heart rate) was less than 5%.

Following these validation checks, all 2,270 participants were retained for descriptive and prevalence analyses (Section 3.1). For multivariable regression models, complete-case analysis yielded an analytic sample of 2,194 participants with non-missing values for all core variables; this sample was used consistently across Sections 3.2–3.7. Subsamples were utilised only when required by specific analytic designs. Specifically, lipid-pathway models (Section 3.8) were estimated among 2,182 participants with complete lipid measurements and covariates, and the sensitivity analysis excluding antihypertensive medication users (Section 3.6.2) was conducted among 1,895 participants reporting no current antihypertensive medication use.

Written informed consent was obtained from all participants at the time of the original health examination, in accordance with national regulations on public health services (31). The secondary analysis of the fully anonymised dataset was approved by the Ethics Committee of Henan University (HU-REC-2025-011) and complied with the Declaration of Helsinki (2013 revision) (27).

### 2.3 Variables and measurements

The variables in this study spanned three primary domains: behavioural exposures (physical activity and alcohol use), cardiovascular and metabolic outcomes, and demographic and clinical covariates. All data were extracted from standardised national health examination forms and cross-referenced with clinical laboratory records from the 2025 Rural Elderly Health Examination Programme (32).

#### 2.3.1 Behavioural exposures

Occupational (labour-type) physical activity (OPA). OPA was assessed using a standardised examination item: “Do you regularly engage in physical exercise or physical activity (including walking, farming, or domestic labour) for ≥30 min each time?” Participants reported the number of such activity sessions per week (sessions/week). This variable was treated as continuous in all primary analyses. For descriptive statistics, OPA was categorised as inactive (0 sessions/week), low (1–2 sessions/week), and moderate-to-high (≥3 sessions/week), following cut-offs consistent with national recommendations for older adults and rural activity profiles in China (33). For sensitivity and joint-exposure analyses, OPA was further operationalised as an ordinal variable and a binary indicator distinguishing lower-frequency (0–2 sessions/week) from high-frequency (≥3 sessions/week) activity. These categorical thresholds were specified *a priori* to enhance interpretability while maintaining adequate cell sizes for stable estimation in this rural cohort. In addition, the substantive inferences were verified using continuous (per-session) models and alternative operationalisations (ordinal and binary) in sensitivity and joint-exposure analyses, yielding materially consistent conclusions.

Alcohol consumption. Alcohol use was evaluated using self-reported drinking frequency (times/week) and beverage type. In this cohort, intake predominantly consisted of high-proof Chinese baijiu (a traditional distilled grain spirit; commonly ~35–60% alcohol by volume [ABV]) consumed within social contexts. Drinking frequency over the preceding 12 months was categorised as never/rare (<1 episode/month), occasional (1–3 episodes/month), and weekly (≥1 occasion/week), adhering to standard epidemiological definitions. In primary models, drinking frequency was treated as a continuous exposure (per +1 occasion/week). For joint-exposure and PAF analyses, drinking was additionally operationalised as none versus any weekly drinking (>0/week) to support interpretable contrasts and ensure robust estimation in fully adjusted models. When necessary, intake was approximated as grams of ethanol using typical conversions for baijiu, assuming ~50 mL per occasion at ~50% alcohol by volume (≈20 g ethanol per occasion) (34).

#### 2.3.2 Cardiovascular and metabolic outcomes

The primary outcome was hypertension, defined as higher-arm systolic blood pressure (SBP) ≥ 140 mmHg and/or diastolic blood pressure (DBP) ≥ 90 mmHg. This definition aligns with Chinese, European, and International Society of Hypertension guidelines and applies the higher-arm principle to enhance epidemiological accuracy (35). Antihypertensive medication status was recorded and reserved for sensitivity analyses.

Secondary cardiometabolic outcomes included dyslipidaemia (TG ≥ 2.3 mmol/L and/or HDL-C < 1.0 mmol/L) (36, 37), impaired fasting glucose (FBG ≥ 6.1 mmol/L) (37, 38), and obesity (BMI ≥ 28 kg/m^2^) (38). Continuous markers (SBP, DBP, TG, LDL-C, HDL-C, FBG, and BMI) were also analysed as secondary outcomes. An exploratory composite cardiometabolic risk score (0–4) was created for descriptive purposes but was not used in primary models (Tables 1–7).

**Table 1:** Baseline characteristics of the study sample by sex (validated dataset).

Variable	Men (*n* = 1,107)	Women (*n* = 1,163)	*p*-value
Age (years)	72.9 ± 6.1	72.5 ± 5.9	0.204
BMI (kg/m^2^)	24.5 ± 3.3	24.5 ± 3.4	0.843
Labour-type physical activity frequency (sessions/week)	1.8 ± 1.5	1.8 ± 1.5	0.823
Alcohol drinking frequency (times/week)	0.40 ± 1.00	0.00 ± 0.30	<0.001
Systolic blood pressure, higher arm (mmHg)	145.6 ± 21.0	145.5 ± 20.4	0.875
Diastolic blood pressure, higher arm (mmHg)	88.0 ± 11.9	85.4 ± 10.7	<0.001
Resting heart rate, winsorized (bpm)	72.8 ± 11.4	72.7 ± 10.8	0.804
Haemoglobin (g/L)	144.7 ± 14.7	132.1 ± 11.0	<0.001
Triglycerides (mmol/L)	1.70 ± 1.05	2.04 ± 1.17	<0.001
Hypertension (SBP ≥ 140 and/or DBP ≥ 90), n (%)	700 (63.3%)	711 (61.1%)	0.290

Continuous variables are presented as mean ± SD and compared between men and women using Welch’s *t*-tests. Categorical variables are presented as *n* (%) and compared using Pearson’s chi-square tests; Fisher’s exact tests were applied when expected cell counts were <5. Hypertension was defined as higher-arm systolic blood pressure (SBP) ≥ 140 mmHg and/or diastolic blood pressure (DBP) ≥ 90 mmHg. Resting heart rate was winsorized at the 1st–99th percentiles to reduce the influence of extreme outliers. Descriptive statistics used all available observations for each variable (pairwise deletion). Missingness for core variables used in the primary analysis pipeline was <5%; no imputation was performed.

**Table 2:** Multivariable logistic regression of physical activity and alcohol consumption with measured hypertension.

Variable	Adjusted OR (95% CI)	*p*-value	Interpretation
Alcohol drinking frequency (per +1 time/week)	1.20 (1.04–1.40)	0.014	Higher drinking frequency increased hypertension risk
Physical activity frequency (per +1 session/week)	1.23 (1.16–1.32)	<0.001	More frequent PA (manual labour) increased hypertension risk

All models were adjusted for age, sex, BMI, haemoglobin concentration, and winsorized resting heart rate, with HC3 heteroscedasticity-consistent standard errors. Labour-type physical activity (e.g., farming/domestic labour) was distinguished from leisure-time exercise. Model diagnostics indicated acceptable calibration (Hosmer–Lemeshow *p* = 0.49) and no evidence of problematic collinearity (VIF 1.07–1.33).

**Table 3:** Dose–response relationships between physical activity, alcohol consumption, and hypertension.

Exposure variable	Test for nonlinearity (*p*)	Linear trend OR (per +1 unit)	95% CI	*p*-value	Interpretation
Physical activity frequency (sessions/week)	0.25	1.23	1.16–1.32	<0.001	Linear increase; no J-shaped pattern
Alcohol drinking frequency (times/week)	0.66	1.20	1.04–1.40	0.014	Monotonic risk increase with drinking

Polynomial extensions of the logistic regression models were fitted to assess potential nonlinearity in the associations of labour-type physical activity (LPA) frequency and alcohol drinking frequency with hypertension. Likelihood ratio tests comparing polynomial versus linear specifications showed no improvement in model fit, supporting approximately linear and monotonic dose–response relationships without evidence of thresholds. All models were adjusted for age, sex, BMI, haemoglobin concentration, and winsorized resting heart rate.

**Table 4:** Population attributable fractions (PAF) of behavioural exposures for hypertension.

Exposure	Exposure prevalence (Pe)	Adjusted OR (95% CI)	PAF (%)	Interpretation
High-frequency physical activity (≥3/week)	59.4%	1.90 (1.57–2.29)	34.8%	≈35% of hypertension cases attributable to heavy occupational labour
Regular drinking (>0/week)	7.9%	1.62 (1.11–2.36)	4.7%	≈5% of cases attributable to habitual weekly drinking
Joint exposure (high PA + regular drinking)	5.7%	1.71 (1.11–2.67)	3.9%	Additional burden from concurrent heavy labour and drinking

Population attributable fractions (PAF) were calculated using Levin’s formula to estimate the proportion of hypertension cases attributable to each exposure. High-frequency labour-type physical activity (≥3 sessions/week) was defined as manual agricultural or domestic labour and interpreted as non-volitional workload rather than leisure-time exercise. The adjusted ORs suggest that a meaningful share of hypertension burden in this cohort may be preventable through workload modification and reduction of weekly alcohol use; the estimated PAF were 34.8% for high-frequency labour-type physical activity and 4.7% for any weekly drinking. All models were adjusted for age, sex, BMI, haemoglobin concentration, and winsorized resting heart rate.

**Table 5:** Sensitivity and validation analyses under alternative hypertension definitions.

Analysis domain	Test/outcome	Key result	Interpretation
Measurement validity	Missingness (SBP/DBP)	<0.2%	Excellent completeness
	Left–right SBP difference	−0.6 ± 8.1 mmHg	No systematic arm bias
	Left–right DBP difference	−0.4 ± 4.9 mmHg	No systematic arm bias
Alternative definitions	Higher-arm ≥140/90	PA 1.23; Alcohol 1.20	Primary definition confirmed; both exposures associated
	Mean-arm ≥140/90	PA 1.20; Alcohol 1.21	Associations robust across arm definitions
	Higher-arm ≥130/80	PA 1.17; Alcohol 1.17	PA remains significant; alcohol effect attenuated at lower threshold

The primary definition of hypertension was based on the higher-arm measurement (SBP ≥140 mmHg and/or DBP ≥90 mmHg). For robustness, we also evaluated alternative definitions using the mean of both arms (≥140/90 mmHg) and a lower higher-arm threshold (≥130/80 mmHg). Labour-type physical activity remained significantly associated with hypertension across all definitions, whereas the alcohol–hypertension association was more sensitive to the lower 130/80 mmHg threshold. Model calibration was acceptable for all specifications (Hosmer–Lemeshow *p* > 0.30). Sensitivity analyses excluding participants who reported current antihypertensive medication use yielded consistent findings.

**Table 6:** Sensitivity analyses of the associations between physical activity, drinking frequency, and hypertension.

Model specification	*N*	OPA OR	Alcohol OR	Interpretation
Model 1 (age, sex, BMI only)	2,194	1.26	1.23	Effects persist under minimal adjustment
Model 2 (physiological covariates added)	2,194	1.23	1.20	Robust to covariate substitution
Model 3 (final multivariable model)	2,194	1.23	1.20	Primary adjusted estimate
Excluding antihypertensive users	1,895	1.20	1.24	Effects persist when treatment bias is removed

All analyses used HC3 heteroscedasticity-consistent standard errors. The final model adjusted for age, sex, BMI, haemoglobin concentration, and winsorized resting heart rate, while minimally adjusted models included age, sex, and BMI only. Excluding participants who reported current antihypertensive medication use did not materially change the estimates, indicating that medication use did not account for the observed associations of labour-type physical activity and alcohol drinking frequency with hypertension.

**Table 7:** Subgroup analyses of the associations between weekly physical activity frequency, alcohol drinking frequency, and measured hypertension.

Subgroup	OPA OR (95% CI)	Alcohol OR (95% CI)	Interpretation
Men	1.27 (1.16–1.40)	1.25 (1.07–1.47)	Strong effects for both exposures among men
Women	1.20 (1.10–1.31)	0.76 (0.48–1.21)	Alcohol not associated with hypertension in women
<70 years	1.08 (0.97–1.20)	1.30 (1.04–1.63)	Alcohol effect stronger in younger older adults
≥70 years	1.32 (1.22–1.44)	1.12 (0.92–1.37)	PA effect strongest in older adults
BMI < 24 kg/m^2^	1.28 (1.17–1.41)	1.37 (1.05–1.78)	Both exposures significantly associated
BMI 24–28 kg/m^2^	1.20 (1.09–1.34)	1.16 (0.94–1.42)	Moderate associations, alcohol non-significant
BMI ≥ 28 kg/m^2^	1.03 (0.84–1.26)	1.00 (0.66–1.49)	No significant associations in high-BMI group

Stratified analyses by sex, age, and BMI indicated meaningful heterogeneity in the associations of labour-type physical activity and alcohol drinking frequency with hypertension. Alcohol drinking frequency was positively associated with hypertension in men but not in women. The labour-type physical activity association was stronger among older adults (≥70 years), whereas the alcohol association was more evident among adults aged <70 years. By BMI category, the strongest associations for both exposures were observed in leaner individuals (BMI <24 kg/m^2^), while neither exposure was significantly associated with hypertension among participants with BMI ≥28 kg/m^2^.

#### 2.3.3 Physiological and laboratory measurements

All physiological assessments followed standardised national protocols for older adults health examinations (39). Bilateral blood pressure was measured after at least 5 min of seated rest using a calibrated Omron HBP-1300 professional electronic sphygmomanometer, validated for use in Chinese populations. Measurements were obtained at heart level with an appropriately sized cuff, and higher-arm values were utilised for primary analyses (35). Height and weight were recorded using a certified stadiometer and electronic scale with participants in light clothing and no shoes, from which body mass index (BMI) was calculated.

Fasting venous blood samples were collected after a minimum 8-h fast and transported under cold-chain conditions for processing at the Wangkui County Central Clinical Laboratory. The biochemical panel included triglycerides, HDL-C, LDL-C, fasting blood glucose, haemoglobin, and routine blood parameters. Automated analysers were operated with daily internal quality control and external proficiency testing, consistent with provincially accredited quality-control processes. Duplicate assays in 10% of participants demonstrated high reliability, with intraclass correlation coefficients exceeding 0.95. Resting heart rate, recorded immediately after blood pressure measurement, was winsorised at the 1st and 99th percentiles to mitigate the influence of extreme values (40).

#### 2.3.4 Covariates

Multivariable models were adjusted for a pre-specified set of clinically relevant covariates: age, sex, BMI, haemoglobin concentration, and winsorised resting heart rate (40). This core adjustment set was selected *a priori* to address major demographic and physiological confounders while ensuring model parsimony. Additional factors (e.g., dietary habits, smoking, and medication use) were incorporated into supplementary analyses (Section 3.6) to evaluate the robustness of the primary findings without materially expanding the main models.

### 2.4 Statistical analysis

All statistical analyses were conducted using R version 4.5.0 (R Foundation for Statistical Computing, Vienna, Austria). The analytic strategy adhered to contemporary cardiovascular epidemiology standards and followed STROBE reporting guidelines (41). The statistical workflow integrated data inspection and preprocessing, primary multivariable regression modelling, and dose–response evaluation. To ensure full analytic traceability and reproducibility, all derived variables—including higher-arm blood pressure, hypertension definitions, winsorised resting heart rate, and recoded exposure variables—were generated using a single reproducible script.

#### 2.4.1 Data inspection and preprocessing

Initial data inspection assessed completeness, physiological plausibility, and distributional characteristics using descriptive statistics, histograms, Q–Q plots, and Shapiro–Wilk tests. Implausible physiological values were screened against age-appropriate clinical reference ranges (42). Given that the clinical dataset had already undergone routine quality checks by local health authorities, no additional exclusions were applied beyond these pre-specified criteria. Resting heart rate occasionally exhibited extreme values and was therefore winsorised at the 1st and 99th percentiles to mitigate undue leverage on regression estimates. All other continuous variables (SBP/DBP, triglycerides, HDL-C, LDL-C, fasting glucose, and BMI) were analysed on their original scale in primary models, with alternative transformations or truncation rules examined only in sensitivity analyses.

Missingness in core analytic variables (blood pressure, OPA, alcohol use, BMI, haemoglobin, and resting heart rate) was less than 5%. Consequently, descriptive and prevalence analyses (Section 3.1) utilised the full sample (*N* = 2,270), whereas primary regression models (Sections 3.2–3.7) employed complete-case data (*N* = 2,194) to ensure consistent covariate adjustment. Multiple imputation was not performed because the low missingness rate allowed complete-case analysis to provide a transparent and consistent modelling sample; robustness was further supported by sensitivity analyses yielding estimates consistent with the primary results.

#### 2.4.2 Primary regression models

The primary outcome was hypertension, defined as higher-arm SBP ≥ 140 mmHg and/or DBP ≥ 90 mmHg, consistent with guideline recommendations (43, 44). Antihypertensive medication status was recorded but was incorporated only in supplementary analyses rather than the primary outcome definition. The main exposures were OPA frequency (sessions/week) and alcohol drinking frequency (occasions/week), both modelled as continuous variables with effects interpreted per +1 unit/week. In this rural context, reported physical activity predominantly reflected non-volitional occupational workload (e.g., farming and domestic labour) rather than leisure-time exercise, and was interpreted accordingly.

Associations between behavioural exposures and hypertension were estimated using multivariable logistic regression with HC3 heteroscedasticity-consistent standard errors (45). Two-sided *p*-values were reported and statistical significance was defined *a priori* as *p* < 0.05. All primary models adjusted for a pre-specified covariate set selected a priori based on biological relevance and prior evidence: age, sex, BMI, haemoglobin concentration, and winsorised resting heart rate. No automated variable selection procedures were used to avoid data-driven model specification. Adjusted odds ratios (aORs) with 95% confidence intervals were reported. The population-attributable fraction (PAF) for selected dichotomised exposures was estimated using Levin’s formula (Section 3.4).

Model stability and fit were evaluated using standard diagnostics. Multicollinearity was assessed with variance inflation factors (VIFs). Calibration was examined using the Hosmer–Lemeshow test based on deciles of predicted risk (*g* = 10). Influential observations were assessed using Cook’s distance, and linearity of the logit for continuous predictors was examined using Box–Tidwell-type log–product terms where applicable.

#### 2.4.3 Dose–response, joint-exposure, and subgroup analyses

Dose–response relationships were evaluated by modelling OPA and alcohol frequency as continuous exposures. To assess potential nonlinearity, polynomial terms and restricted cubic splines were fitted and compared against linear specifications using likelihood ratio tests. To preserve interpretability and reduce overfitting risk, linear functional forms were retained unless model-comparison tests provided evidence of a materially superior fit for nonlinear specifications.

Combined exposure patterns were evaluated using a joint-exposure approach in which OPA was dichotomised as lower-frequency (0–2 sessions/week) versus high-frequency (≥3 sessions/week), and alcohol use as non-drinking (0 occasions/week) versus regular drinking (≥1 occasion/week). Four mutually exclusive groups were constructed, with the lower-frequency OPA/non-drinking group specified *a priori* as the referent. Effect heterogeneity was explored through pre-specified subgroup analyses stratified by sex, age (<70 vs. ≥70 years), and BMI category (<24, 24–28, and ≥28 kg/m^2^). Stratified models used the same adjustment set as the primary model, except that the stratifying variable was not additionally adjusted for in its corresponding stratum, and HC3 standard errors were applied throughout to maintain comparability.

#### 2.4.4 Sensitivity and robustness analyses

A comprehensive set of sensitivity analyses was conducted to evaluate the internal validity of the primary findings. We examined the robustness of the estimates to alternative hypertension definitions (mean-arm ≥140/90 mmHg and higher-arm ≥130/80 mmHg) and to the exclusion of participants reporting current antihypertensive medication use. We also assessed whether results were sensitive to outlier-handling strategies for behavioural exposures (winsorisation and upper-tail trimming) and to covariate specification using nested adjustment sets (minimal, physiological, and fully adjusted). Additional models included SBP-only and DBP-only outcome definitions, linear models for continuous SBP and DBP, and multiplicative interaction testing between OPA and alcohol frequency. Across these analyses, the direction and magnitude of associations were materially unchanged, supporting the robustness of the primary conclusions.

### 2.5 Ethical approval and data governance

All study procedures conformed to the ethical principles of the Declaration of Helsinki (2013 revision) and national regulations governing the management of health examination data for older adults in China. The 2025 Rural Elderly Health Examination Programme was organised by the Wangkui County Health Bureau and implemented by the Wangkui County Centre for Disease Control and Prevention (CDC) as part of routine public health services. During the initial examination, all participants were informed of the purpose and procedures, as well as the potential use of anonymised health examination data for scientific research. Written informed consent was obtained by trained clinical staff at the point of examination.

Clinical and laboratory data—including bilateral blood pressure, anthropometric measurements, fasting venous blood collection, and biochemical testing—were recorded and stored in accordance with the National Basic Public Health Service Specification (2023 Edition) and provincial guidelines for health data management. In November 2025, the Wangkui County Health Bureau formally authorised Henan University to conduct a secondary scientific analysis of the fully anonymised dataset. All personal identifiers, including names, identification numbers, addresses, and telephone numbers, were removed prior to data transfer. The dataset was encrypted and access was restricted to authorised study personnel in accordance with applicable data security and governance requirements.

The secondary analysis protocol was reviewed and approved by the Henan University Biomedical Research Ethics Sub-Committee (approval no. HUSOM2025-929). Given that the dataset was fully anonymised and derived from routine public health services, the requirement for additional informed consent for the secondary analysis was waived. The study was conducted and reported in accordance with STROBE guidelines, ICMJE recommendations, and COPE principles for research integrity.

## 3 Results

### 3.1 Baseline characteristics

A total of 2,270 community-dwelling older adults were included in the validated analytic dataset, comprising 1,107 men (48.8%) and 1,163 women (51.2%). Blood pressure was measured bilaterally, and the higher-arm value was used for primary analyses to avoid underestimation of vascular risk. The overall systolic/diastolic blood pressure (SBP/DBP) was 145.5 ± 20.7/86.7 ± 11.4 mmHg, indicating a high vascular load in this rural older-adult sample.

In sex-stratified comparisons, SBP did not differ between men and women (145.6 ± 21.0 vs. 145.5 ± 20.4 mmHg; *p* = 0.875), whereas DBP was higher in men (88.0 ± 11.9 vs. 85.4 ± 10.7 mmHg; *p* < 0.001). After winsorization at the 1st–99th percentiles, the mean resting heart rate was 72.7 ± 11.1 bpm, with no evidence of a sex difference (men: 72.8 ± 11.4 bpm; women: 72.7 ± 10.8 bpm; *p* = 0.804).

Haemoglobin concentrations were higher in men than in women (144.7 ± 14.7 vs. 132.1 ± 11.0 g/L; *p* < 0.001). Conversely, triglyceride levels were higher in women (2.04 ± 1.17 vs. 1.70 ± 1.05 mmol/L; *p* < 0.001).

Using measurement-based criteria (higher-arm SBP ≥ 140 mmHg and/or DBP ≥ 90 mmHg), the prevalence of hypertension was 62.2% overall. Although prevalence was numerically higher in men (63.3%) than in women (61.1%), the sex difference was not statistically significant (*p* = 0.290).

### 3.2 Association between physical activity, alcohol consumption, and hypertension

Multivariable logistic regression was used to examine the associations of weekly OPA frequency and alcohol drinking frequency with measured hypertension. Physical activity was defined as the number of labour-type physical activity per week, and alcohol exposure as the number of drinking occasions per week. All models were adjusted for age, sex, body mass index (BMI), haemoglobin concentration, and winsorized resting heart rate.

**Figure 3:**
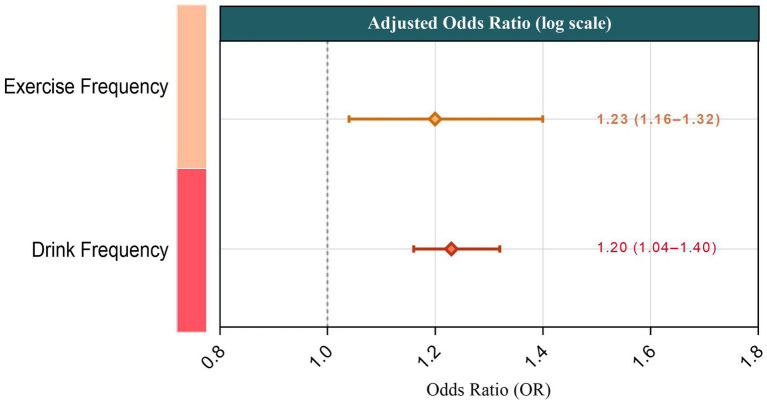
Adjusted odds ratios (OR) and 95% confidence intervals (CI) for the association between physical activity frequency and alcohol consumption frequency with hypertension in older adults. The figure illustrates the positive association between both physical activity frequency (OR = 1.23, 95% CI: 1.16–1.32) and alcohol drinking frequency (OR = 1.20, 95% CI: 1.04–1.40) and the increased likelihood of hypertension, adjusted for age, sex, body mass index (BMI), haemoglobin concentration, and resting heart rate.

Each additional PA session per week was associated with higher odds of hypertension (adjusted OR = 1.23, 95% CI: 1.16–1.32; *p* < 0.001). Similarly, each additional drinking occasion per week increased the likelihood of hypertension (adjusted OR = 1.20, 95% CI: 1.04–1.40; *p* = 0.014). The positive association between both physical activity and alcohol consumption and increased risk of hypertension can be clearly seen in Figure 3, which presents the adjusted odds ratios (OR) along with 95% confidence intervals (CI) for both exposures.

Given that “physical activity” in this rural older adults cohort predominantly reflects manual or agricultural labour rather than structured leisure-time exercise, the positive association likely reflects heavy occupational workload and insufficient recovery, rather than health-promoting activity. Figure 3 helps illustrate this by showing the adjusted odds ratio for physical activity frequency (OR = 1.23) and alcohol drinking frequency (OR = 1.20) with their respective confidence intervals.

Model diagnostics indicated acceptable calibration (Hosmer–Lemeshow *p* = 0.49) and no evidence of problematic collinearity (all VIF values ≈ 1.07–1.33). Pairwise correlations among predictors were low (|*r*| generally 0.00–0.44), supporting model stability.

### 3.3 Dose–response and nonlinear trend analysis

To examine whether the relationships between weekly labour-type physical activity (LPA), alcohol consumption, and hypertension deviated from linearity, polynomial extensions of the logistic models were fitted while maintaining identical covariate adjustments to the main analytic pipeline.

#### 3.3.1 Physical activity frequency

Quadratic and cubic terms were added to the PA model. The likelihood ratio test (LRT) comparing the linear and polynomial models indicated no improvement in model fit (*χ*^2^ = 1.31, df = 1, *p* = 0.25), suggesting no evidence for a J-shaped, U-shaped, or threshold pattern.

The linear dose–response association remained significant. Each additional weekly PA session was associated with 23% higher odds of hypertension (adjusted OR = 1.23, 95% CI: 1.16–1.32; *p* < 0.001).

**Figure 4:**
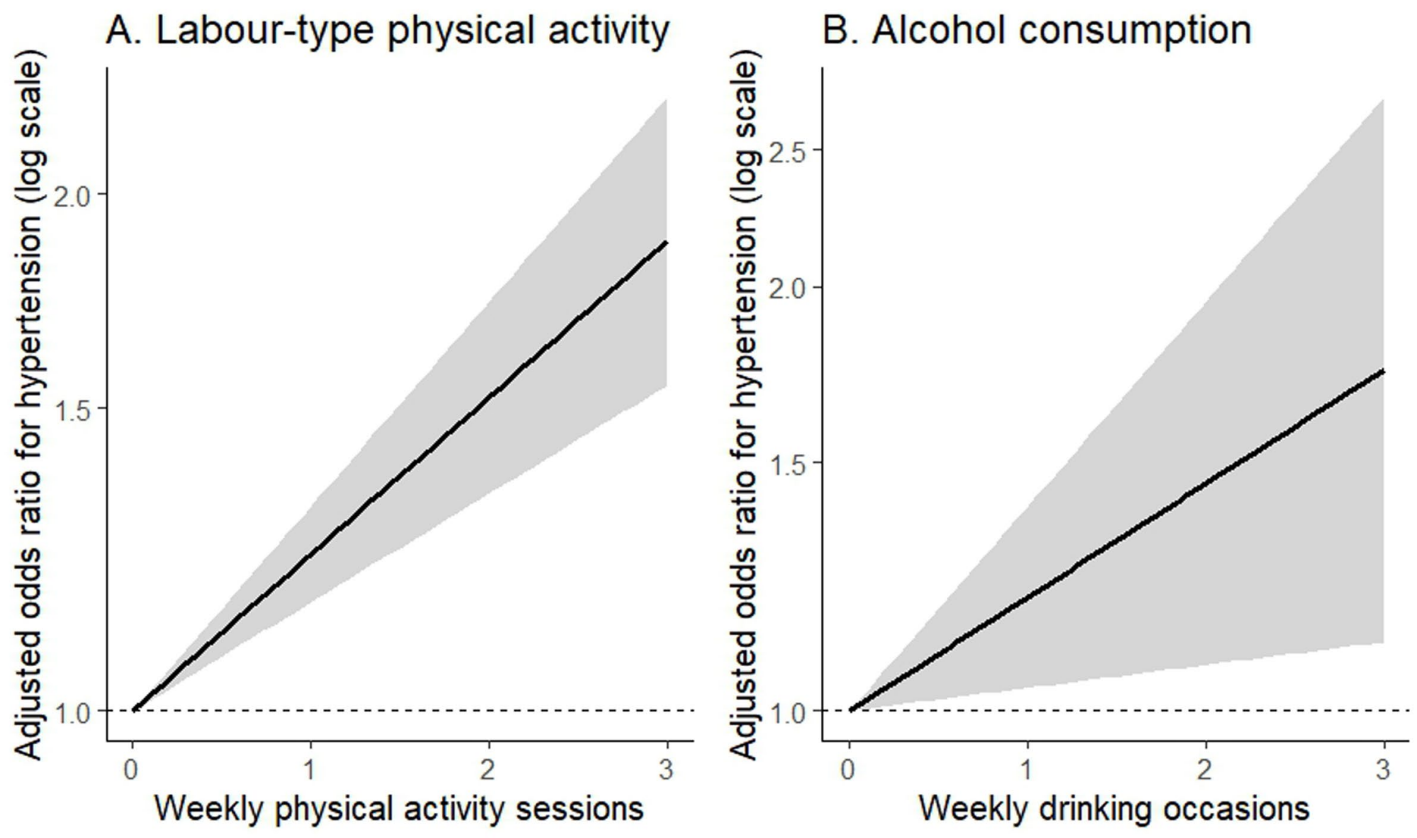
Dose–response relationships between weekly physical activity, alcohol consumption, and hypertension. **(A)** Linear trend of adjusted odds ratios for hypertension across weekly labour-type physical activity sessions. **(B)** Linear trend of adjusted odds ratios across weekly drinking occasions.

The predicted linear trend and 95% CI band are displayed in Figure 4A.

#### 3.3.2 Alcohol consumption frequency

Similarly, polynomial models for alcohol drinking frequency showed no meaningful improvement over the linear specification (LRT *χ*^2^ = 0.20, df = 1, *p* = 0.66).

Each additional weekly drinking occasion was associated with 20% higher odds of hypertension (adjusted OR = 1.20, 95% CI: 1.04–1.40; *p* = 0.014).

The estimated linear trend is illustrated in Figure 4B.

#### 3.3.3 Summary

Together, these analyses support approximately linear, monotonic exposure–response relationships for both labour-type physical activity and alcohol frequency. Hypertension risk increased steadily with higher LPA frequency, without evidence of a protective range at lower exposure levels. Alcohol frequency showed a similarly monotonic gradient, with no detectable plateau.

**Figure 5:**
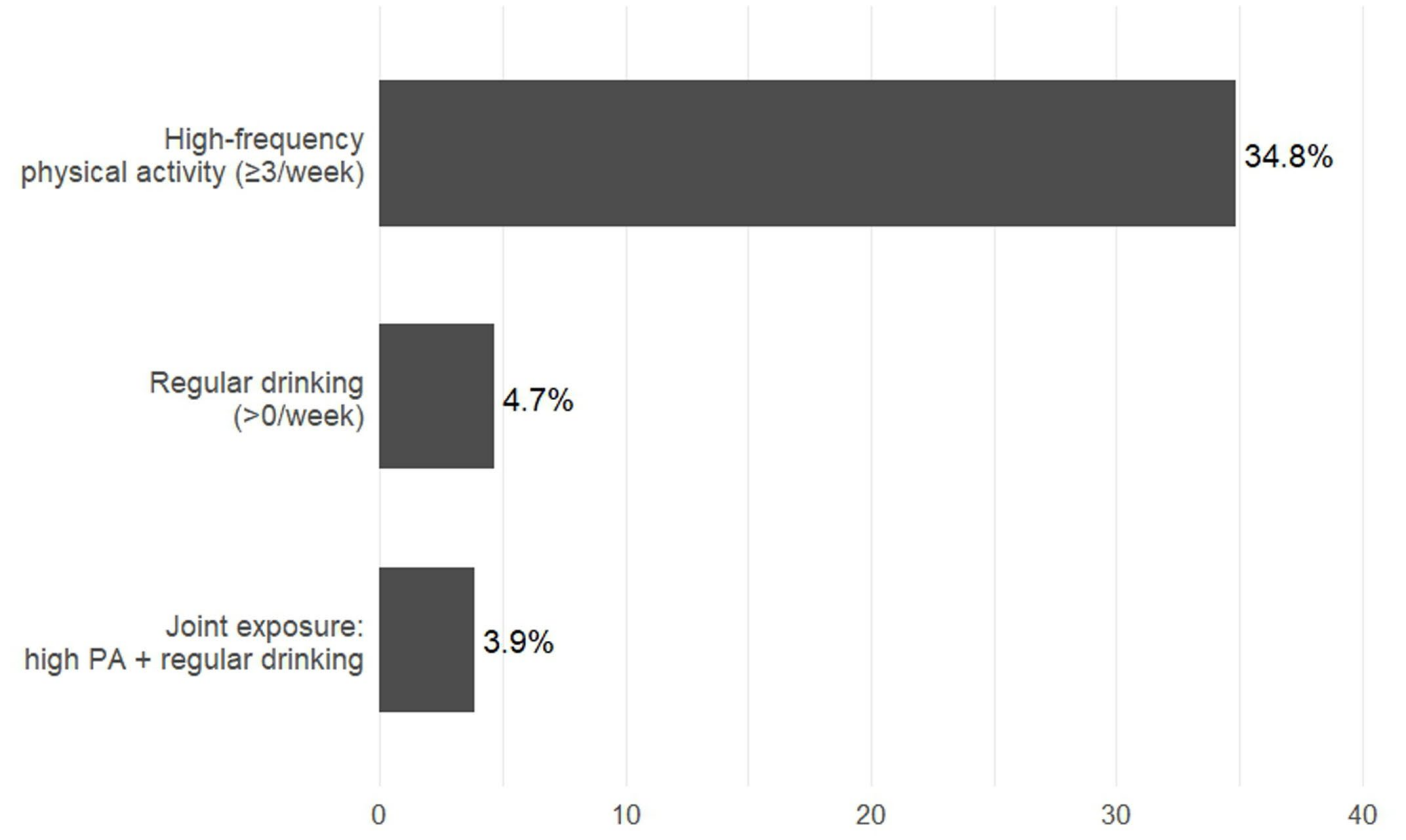
Population attributable fraction (PAF) for hypertension by behavioural exposure. The bars illustrate the estimated PAF for high-frequency labour-type physical activity of at least three sessions per week, any weekly alcohol drinking, and joint exposure to both high-frequency labour-type physical activity and weekly drinking. The corresponding PAF were 34.8, 4.7, and 3.9%, respectively.

Shaded areas represent 95% confidence intervals generated from logistic regression models with HC3 robust standard errors. All models were adjusted for age, sex, BMI, haemoglobin concentration, and winsorized resting heart rate. Nonlinearity tests (polynomial LRTs) indicated no significant deviation from linearity for either exposure (Figure 5).

### 3.4 Population-attributable risk and public health implications

To quantify the population-level burden associated with key behavioural exposures, population-attributable risks (PAF) were estimated using exposure prevalences from the validated dataset and adjusted odds ratios (ORs) derived from multivariable logistic regression models. PAF were calculated using Levin’s formula:


$$\mathrm{PAF}=\frac{P_{e}(\text{OR}-1)}{P_{e}(\text{OR}-1)+1}$$

#### 3.4.1 High-frequency physical activity

High-frequency physical activity (≥3 sessions per week)—which in this context largely reflects manual agricultural or domestic labour—was highly prevalent, with 59.4% of Participants reporting such activity. Compared with lower activity levels, high-frequency physical activity was associated with a substantially higher risk of hypertension (adjusted OR = 1.90, 95% CI: 1.57–2.29).

The corresponding PAF was 34.8%, indicating that approximately one in three hypertension cases could theoretically be prevented if heavy, non-recoverable physical labour were replaced with safer and more sustainable activity patterns.

#### 3.4.2 Regular drinking

Regular drinking—defined operationally as any weekly drinking (>0 drinking sessions per week)—was reported by 7.9% of Participants. Despite its relatively low prevalence, habitual weekly alcohol consumption was associated with an elevated risk of hypertension (adjusted OR = 1.62, 95% CI: 1.11–2.36).

The corresponding PAF was 4.7%, suggesting that approximately one in 20 hypertension cases in this population may be attributable to weekly alcohol use.

#### 3.4.3 Joint exposure to high-frequency physical activity and regular drinking

Joint exposure to both high-frequency physical activity and regular drinking was present in 5.7% of Participants. This combined exposure was associated with a higher risk of hypertension (adjusted OR = 1.71, 95% CI: 1.11–2.67).

The corresponding PAF was 3.9%, reflecting an additional but modest population-level burden arising from the co-occurrence of heavy occupational workload and habitual drinking.

#### 3.4.4 Public health implications

Collectively, the PAF estimates suggest that a substantial proportion of hypertension cases in this rural older adults population may be attributable to heavy occupational labour and habitual weekly drinking. Importantly, in this setting, “physical activity” represents physically demanding, non-volitional labour, rather than structured, moderate-intensity exercise typically promoted in clinical guidelines.

Public health strategies should therefore emphasise workload modulation (e.g., mechanisation, task redistribution, rest scheduling) and alcohol-use reduction, rather than simply encouraging higher overall volumes of physical activity.

### 3.5 Measurement validation and alternative hypertension definitions

To evaluate the robustness of the observed associations, we conducted both measurement-level and definition-level validation analyses.

#### 3.5.1 Measurement validity

Bilateral blood pressure measurements showed excellent completeness, with missingness <0.2% for all four arm-specific variables. Left–right differences were small and symmetric, with a mean (±SD) of −0.6 ± 8.1 mmHg for systolic blood pressure (SBP) and −0.4 ± 4.9 mmHg for diastolic blood pressure (DBP). These findings indicate no systematic arm-specific measurement bias and support the use of the higher-arm reading as the primary criterion for hypertension classification, consistent with AHA and BHS recommendations.

#### 3.5.2 Alternative hypertension definitions

To assess whether the associations between behavioural factors (weekly physical activity frequency and drinking frequency) and hypertension were sensitive to outcome definition, multivariable logistic regression models were re-estimated under three commonly used blood pressure thresholds. All models were adjusted for age, sex, BMI, haemoglobin concentration, and winsorized resting heart rate.

When hypertension was defined as higher-arm BP ≥ 140/90 mmHg (primary definition), physical activity frequency remained strongly associated with hypertension (OR = 1.23, 95% CI: 1.16–1.32), and drinking frequency also showed a positive association (OR = 1.20, 95% CI: 1.04–1.40). Model calibration was acceptable (Hosmer–Lemeshow *p* = 0.49).

Using the mean of left- and right-arm readings (mean-arm ≥140/90 mmHg) produced very similar effect estimates (physical activity: OR = 1.20, 95% CI: 1.13–1.27; alcohol: OR = 1.21, 95% CI: 1.05–1.39), with good calibration (Hosmer–Lemeshow *p* = 0.41).

When a lower diagnostic threshold of higher-arm BP ≥ 130/80 mmHg was applied, the association with physical activity persisted (OR = 1.17, 95% CI: 1.05–1.29), whereas the alcohol–hypertension association was weaker and not statistically significant (OR = 1.17, 95% CI: 0.91–1.50). Calibration remained acceptable (Hosmer–Lemeshow *p* = 0.34).

Taken together, these analyses show that physical activity frequency is a stable predictor of hypertension across multiple blood pressure definitions (OR range: 1.17–1.23). The association with drinking frequency is directionally consistent but more sensitive to the specific threshold used, becoming non-significant at the lower 130/80 mmHg cut-off. Acceptable goodness-of-fit across all models supports the internal validity of these findings.

### 3.6 Sensitivity analyses for covariate specification and treatment status

To assess the robustness of the associations between behavioural exposures and measured hypertension, we conducted a series of model-based sensitivity analyses focusing on covariate specification and the exclusion of Participants receiving antihypertensive treatment. All models used HC3 heteroscedasticity-consistent standard errors.

#### 3.6.1 Covariate specification

First, three progressively adjusted logistic regression models were estimated to examine whether the associations of weekly labour-type physical activity (LPA) frequency and drinking frequency with hypertension were sensitive to the choice of covariates.

In the minimally adjusted model including age, sex, and BMI, both exposures showed positive associations with hypertension (OOPA: OR = 1.26; alcohol: OR = 1.23). After adding haemoglobin concentration and winsorized resting heart rate (physiological covariates), the effect sizes were slightly attenuated but remained very similar (OPA: OR = 1.23; alcohol: OR = 1.20). The final multivariable model, which corresponds to the primary specification used in Section 3.2, produced identical estimates (OOPA: OR = 1.23; alcohol: OR = 1.20).

The close agreement of odds ratios across all three models indicates that the observed associations are not artefacts of covariate selection and are unlikely to be explained by either omitted-variable or overadjustment bias.

#### 3.6.2 Exclusion of antihypertensive medication users

To further address potential treatment-related bias, the final multivariable model was re-estimated after excluding all participants who reported current use of antihypertensive medications (*N* = 1,895). In this restricted sample, the associations between behavioural exposures and hypertension remained directionally consistent and of similar magnitude (OOPA: OR = 1.20; alcohol: OR = 1.24).

These findings suggest that the positive associations of OPA frequency and drinking frequency with measured hypertension are not driven by medication-related suppression of blood pressure.

#### 3.6.3 Overall robustness

Across all sensitivity analyses—minimal adjustment, addition of physiological covariates, full adjustment, and exclusion of treated individuals—the associations between behavioural exposures and hypertension were stable in both direction and magnitude. Additional checks using winsorized OPA and alcohol frequencies and excluding the top 5% of exposure values yielded nearly identical odds ratios (data not shown), further confirming the robustness of the main findings.

### 3.7 Interaction and heterogeneity analysis

To examine whether the associations of labour-type physical activity (LPA) frequency and alcohol drinking frequency with hypertension differed across key population subgroups, we performed stratified logistic regression analyses by sex, age group, and BMI category. Within each stratum, models included the same covariates as the primary regression model—age, sex, BMI, haemoglobin concentration, and winsorised resting heart rate—with the exception that the stratifying variable (sex, age group, or BMI category) was not included as a covariate in its corresponding stratified analysis. All models used HC3 heteroscedasticity-consistent standard errors.

#### 3.7.1 Sex-specific heterogeneity

Clear sex differences were observed. Among men, both OPA and alcohol drinking frequency were positively associated with hypertension (OOPA: OR = 1.27, 95% CI: 1.16–1.40; alcohol: OR = 1.25, 95% CI: 1.07–1.47). Among women, OPA remained a significant predictor (OR = 1.20, 95% CI: 1.10–1.31), whereas alcohol consumption showed no significant association with hypertension (OR = 0.76, 95% CI: 0.48–1.21). These patterns suggest that the detrimental effect of alcohol on blood pressure is largely confined to men, while the impact of LPA is present in both sexes but more pronounced in men.

#### 3.7.2 Age-specific heterogeneity

A marked age gradient was also evident.

In Participants aged ≥70 years, OPA frequency showed a strong and statistically significant association with hypertension (OR = 1.32, 95% CI: 1.22–1.44), whereas alcohol use was not significantly related to hypertension (OR = 1.12, 95% CI: 0.92–1.37).

Conversely, among Participants aged <70 years, alcohol had a stronger effect (OR = 1.30, 95% CI: 1.04–1.63), while the association between OPA and hypertension was weaker and not statistically significant (OR = 1.08, 95% CI: 0.97–1.20).

Taken together, these findings indicate that alcohol contributes more to hypertension risk in the younger older adults, whereas occupational workload becomes the dominant behavioural risk factor among adults aged ≥70 years.

#### 3.7.3 BMI-specific heterogeneity

Substantial heterogeneity by BMI category was observed.

Among individuals with BMI < 24 kg/m^2^, both exposures were strong predictors of hypertension (OOPA: OR = 1.28, 95% CI: 1.17–1.41; alcohol: OR = 1.37, 95% CI: 1.05–1.78). In the 24–28 kg/m^2^ group, associations persisted but were weaker, with OPA remaining significant (OR = 1.20, 95% CI: 1.09–1.34) and alcohol showing a non-significant trend (OR = 1.16, 95% CI: 0.94–1.42).

Among Participants with BMI ≥ 28 kg/m^2^, neither OPA nor alcohol was significantly associated with hypertension (OOPA: OR = 1.03, 95% CI: 0.84–1.26; alcohol: OR = 1.00, 95% CI: 0.66–1.49).

These results suggest that leaner individuals (BMI < 24 kg/m^2^) are Particularly susceptible to both occupational physical strain and alcohol-related increases in blood pressure, whereas these behavioural risks appear attenuated in those with higher BMI, potentially reflecting adiposity-related buffering or survivor bias.

#### 3.7.4 Summary of heterogeneity patterns

Across stratified analyses, three consistent patterns emerged. First, alcohol-related hypertension risk was concentrated in men, adults aged <70 years, and leaner individuals (BMI < 24 kg/m^2^). Second, the association between labour-type physical activity and hypertension strengthened with age and was most pronounced among participants aged ≥70 years. Third, among individuals with BMI ≥ 28 kg/m^2^, neither labour-type physical activity nor alcohol drinking frequency was significantly associated with hypertension. Collectively, these findings indicate meaningful effect heterogeneity and underscore the need for population-specific hypertension prevention strategies that account for sex, age, and adiposity profiles.

### 3.8 Metabolic pathway and model diagnostics

To further assess whether behavioural exposures influence hypertension through metabolic pathways, we examined lipid biomarkers—including triglycerides (TG), low-density lipoprotein cholesterol (LDL-C), and high-density lipoprotein cholesterol (HDL-C)—and evaluated the adequacy of the final multivariable model. All analyses used HC3 heteroscedasticity-consistent standard errors and were adjusted for age, sex, BMI, haemoglobin concentration, and winsorized resting heart rate.

#### 3.8.1 Associations between behavioural factors and lipid biomarkers

HC3-robust linear regression models showed no statistically significant associations between weekly labour-type physical activity (LPA) frequency, alcohol drinking frequency, and fasting lipid Parameters after covariate adjustment. Although small coefficients were observed for TG, LDL-C, and HDL-C, none reached conventional significance thresholds (all *p* ≥ 0.05).

These findings indicate that in this rural older adults cohort—where “physical activity” largely reflects heavy manual labour—variation in OPA or drinking frequency does not translate into systematic alterations in TG, LDL-C, or HDL-C at the population level. In other words, the behavioural exposures examined here do not appear to exert their hypertensive effects primarily through fasting lipid profiles (Table 8).

**Table 8:** Associations of physical activity and alcohol drinking frequency with fasting lipid biomarkers (HC3-robust linear regression models).

Biomarker	Predictor	*β* (unstandardized)	*p*-value	Interpretation
TG	Alcohol frequency (+1/week)	Small, ns	≥0.05	No clear dose–response pattern
	Physical activity (+1/week)	Small, ns	≥0.05	No meaningful association
HDL-C	Alcohol frequency	Small, ns	≥0.05	Trend-level but not statistically robust
	Physical activity	Small, ns	≥0.05	No meaningful association
LDL-C	Alcohol frequency	≈0, ns	≥0.10	No consistent relationship
	Physical activity	≈0, ns	≥0.10	No consistent relationship

HC3-robust linear regression models were used to examine associations of labour-type physical activity frequency and alcohol drinking frequency with fasting lipid biomarkers (TG, LDL-C, and HDL-C). Neither behavioural exposure showed a statistically significant association with these lipid measures, suggesting that the observed hypertension associations are more likely to reflect non-lipid pathways (e.g., cumulative haemodynamic stress or neurohumoral activation) in this cohort. All models were adjusted for age, sex, BMI, haemoglobin concentration, and winsorized resting heart rate.

#### 3.8.2 Integrated behavioural–lipid–hypertension models

To assess whether lipid profiles contributed to the association between behavioural exposures and hypertension, triglycerides (TG), LDL-C, and HDL-C were added to the multivariable logistic regression model alongside labour-type physical activity frequency and alcohol drinking frequency (*N* ≈ 2,182). In this extended model, TG was positively associated with hypertension (OR = 1.10 per 1 mmol/L, 95% CI: 1.01–1.21; *p* = 0.032), whereas LDL-C was not (OR = 0.98, 95% CI: 0.88–1.09; *p* = 0.691). HDL-C also showed a positive association with hypertension (OR = 1.76 per 1 mmol/L, 95% CI: 1.26–2.47; *p* = 0.001), which is opposite to the typical cardioprotective expectation and is more plausibly explained by medication use, reverse causation, or survivor bias in an older population than by a causal adverse effect of HDL-C. Importantly, both behavioural exposures remained significant after lipid adjustment, with only modest attenuation (labour-type physical activity: OR = 1.24 per 1 session/week, 95% CI: 1.16–1.32; *p* < 0.001; alcohol drinking frequency: OR = 1.18 per 1 occasion/week, 95% CI: 1.02–1.36; *p* = 0.030). Together, these findings suggest that fasting lipids explain only a small proportion of the observed behavioural associations, and that non-lipid mechanisms—such as cumulative haemodynamic load, neurohumoral activation, and occupational strain—are likely to contribute more substantially (Table 9).

**Table 9:** Multivariable logistic regression of hypertension on behavioural exposures and lipid biomarkers.

Variable	Adjusted OR	95% CI	*p*-value	Interpretation
Physical activity (+1 session/week)	1.24	1.16–1.32	<0.001	Heavy, frequent labour increased hypertension risk
Alcohol drinking frequency (+1 time/week)	1.18	1.02–1.36	0.030	More frequent drinking increased hypertension risk
Triglycerides (per +1 mmol/L)	1.10	1.01–1.21	0.032	Elevated TG modestly increased vascular risk
LDL-C (per +1 mmol/L)	0.98	0.88–1.09	0.691	No independent association
HDL-C (per +1 mmol/L)	1.76	1.26–2.47	0.001	Higher HDL-C associated with higher hypertension risk*

The extended model included labour-type physical activity frequency, alcohol consumption, and lipid biomarkers (TG, LDL-C, and HDL-C). Both physical activity frequency and alcohol drinking frequency remained significant predictors of hypertension, even after adjusting for lipid biomarkers. HDL-C showed an unexpected positive association with hypertension, likely due to medication effects, reverse causation, or survivor bias in this older adults cohort. While triglycerides and HDL-C were significantly associated with hypertension, their contribution to the observed behavioural–hypertension relationship was minimal.

#### 3.8.3 Model diagnostics and assumption checks

Diagnostic checks for the final hypertension model—including OPA, alcohol frequency, and the core covariates—indicated adequate model performance.

Variance inflation factors showed no evidence of problematic multicollinearity (all VIF < 2.0). Influence diagnostics based on Cook’s distance indicated that no observation exerted undue influence on the model: the maximum Cook’s D was well below 0.5, and 0% of observations exceeded conventional thresholds of 0.5 or 1.0.

The model demonstrated acceptable calibration in the Hosmer–Lemeshow goodness-of-fit test (*p* ≈ 0.59), supporting the internal validity of the estimated odds ratios. Deviance residuals were approximately symmetric, with no extreme outliers, indicating no major violations of overall model fit.

Box–Tidwell tests suggested minor departures from the linearity-of-logit assumption for age and resting heart rate, whereas BMI and haemoglobin satisfied linearity. Given the small magnitude of these departures and the stability of the OPA and alcohol odds ratios across multiple sensitivity and subgroup analyses, these deviations were judged unlikely to materially affect the substantive conclusions. Overall, the behavioural–lipid–hypertension models appear statistically robust and consistent with the main findings reported in Sections 3.2–3.7.

## 4 Discussion

This study revealed three interrelated patterns linking occupational (labour-type) physical activity (OPA), alcohol consumption, and hypertension among rural older adults. These findings must be interpreted within the unique environmental and sociocultural context of Northeast China. In this setting, “physical activity” primarily reflects non-volitional, workload-driven occupational demands rather than structured leisure-time exercise. Consequently, the observed associations should be understood in the context of workload-related vascular strain under place-based constraints, rather than assumed to reflect the typical protective effects of voluntary activity reported in urban cohorts. Together, these findings demonstrate that cardiovascular risk in rural ageing populations arises not simply from individual behaviours, but from the complex interaction of community environments, labour demands, and biological stress pathways.

### 4.1 Major findings in the context of rural community environments

Our analysis identified three interconnected behavioural–biological patterns shaping hypertension risk in rural older adults, including a positive linear dose–response association for occupational (labour-type) physical activity (OPA), a clear monotonic alcohol–hypertension association with subgroup-specific vulnerability, and modest yet independent metabolic correlates. These findings must be interpreted within the unique environmental and sociocultural context of Northeast China, where daily behavioural exposures are dictated by structural constraints—including agricultural labour systems, climatic burdens, and entrenched community norms—rather than individual leisure preferences. Collectively, the results indicate that cardiovascular risk in rural ageing populations arises from the interaction of behavioural patterns with place-based environmental pressures, rather than from lifestyle choices alone (6).

First, OPA was positively and linearly associated with hypertension. In contrast to the well-documented protective effects of structured, moderate-intensity leisure-time exercise reported in urban or international cohorts, physical activity in this rural setting primarily involved high-intensity agricultural or manual labour, often protracted and performed in cold temperatures with limited opportunities for recovery (46). Such conditions elicit sustained sympathetic activation, increased vascular load, and cumulative haemodynamic stress. Notably, our supplementary analyses support this interpretation across alternative operationalisations of blood pressure risk: the OPA association remained directionally consistent when hypertension was defined using SBP-only and DBP-only thresholds (Supplementary Table S2), and OPA frequency also showed positive associations with continuous SBP/DBP in HC3-robust linear models (Supplementary Table S3). These findings alleviate concerns that the observed association is an artefact of dichotomising blood pressure at 140/90 mmHg. This labour–hypertension association was most pronounced among adults aged ≥70 years, consistent with the subgroup patterns reported in Section 3.7, underscoring that the cardiovascular implications of ‘physical activity’ are context-dependent and vary substantially by type, intensity, and environmental conditions (47). Furthermore, the age-dependent amplification of the OPA–hypertension association is supported by the OPA × Age interaction model (Supplementary Table S5) and illustrated in Supplementary Figure S1: the predicted probability of hypertension increases most steeply with OPA frequency at older ages (e.g., 85 years), consistent with heightened vulnerability to workload-related haemodynamic strain in advanced age.

Second, alcohol consumption showed a clear monotonic dose–response relationship with hypertension. Regular drinking—defined as any weekly consumption (>0 times/week)—was associated with elevated hypertension risk, even after rigorous multivariable adjustment. High-proof distilled spirits and persistent social drinking norms in rural communities may exacerbate sympathetic activation, oxidative stress, endothelial dysfunction, and altered vascular tone. The alcohol–hypertension association also showed notable subgroup heterogeneity, with more potent effects among men, individuals aged <70 years, and those with a BMI < 24 kg/m^2^, aligning closely with the subgroup results in Section 3.7 (46). These patterns suggest that alcohol-related cardiovascular risk operates within demographic and sociocultural constraints specific to rural settings. However, no clear evidence of multiplicative interaction was found between OPA frequency and alcohol drinking frequency in the supplementary interaction model (OPA × Alcohol term; Supplementary Table S4). Therefore, the joint burden of heavy labour and drinking should be interpreted as potentially cumulative or additive in practice, rather than synergistic on the multiplicative scale.

Third, regarding metabolic markers, while behavioural exposures showed only weak relationships with fasting lipid profiles, triglycerides and HDL-C exhibited modest but independent associations with hypertension. The unexpected positive association between HDL-C and hypertension, also observed in other geriatric cohorts, likely reflects medication effects, reverse causation, or survival bias. Importantly, adjustment for triglycerides, LDL-C, and HDL-C produced minimal attenuation of the behavioural associations, suggesting that lipid alterations explain little of the observed relationships. Instead, non-lipid pathways—including haemodynamic load, neurohumoral activation, cold-weather vascular responses, and cumulative occupational strain—likely contribute more strongly in this context.

Taken together, these findings indicate that health-related behaviours in rural communities should be understood as environmentally conditioned, shaped by cold-climate burdens, agricultural labour structures, community drinking norms, and limited access to preventive care. This interpretation aligns with the WHO Healthy Ageing Framework, which emphasises environmental determinants of intrinsic capacity across the life course. More broadly, the results contribute to place-based cardiovascular epidemiology by illustrating how rural environments influence hypertension risk through interconnected behavioural and physiological pathways. These contextualised findings suggest that hypertension prevention in rural settings should prioritise workload modulation, safer organisation of labour tasks, and reducing any weekly drinking, rather than generic recommendations to ‘increase physical activity’.

### 4.2 Integration with global evidence and theoretical frameworks

These findings align with the Physical Activity Paradox literature, which distinguishes occupational or mandatory activity from leisure-time exercise and emphasises insufficient recovery and sustained haemodynamic load as plausible pathways linking labour-intensive activity to elevated cardiovascular risk (10). Specifically, long-duration, repetitive, and often static occupational tasks may sustain elevated heart rate and blood pressure over prolonged periods, with limited opportunities for rest—conditions typically absent in leisure-time exercise (10). By demonstrating directionally different exposure–response patterns in a resource-limited rural setting, this study extends the global evidence base derived from large-scale cohorts such as PURE, GBD, and the UK Biobank, which predominantly reflect urban or high-income contexts where physical activity is discretionary and recovery-supported (48). In the present cohort, occupational physical activity (OPA) functions as a vascular stressor rather than a protective exposure, highlighting the critical role of task structure, recovery constraints, and sustained physiological load in shaping cardiovascular outcomes (7, 11).

Our results also resonate with the allostatic load framework, which conceptualises repeated behavioural and environmental stressors as sources of cumulative physiological burden (49, 50). The observed linear risk gradients—where each additional unit of OPA or drinking frequency was associated with higher odds of hypertension—are theoretically consistent with cumulative load mechanisms. Although neuroendocrine biomarkers were not measured, the combination of high labour intensity, cold exposure, and limited recovery time characteristic of rural agricultural work plausibly contributes to sustained sympathetic activation and haemodynamic strain. The age-amplified workload association observed in our supplementary analyses (Supplementary Table S5; Supplementary Figure S1) further supports an allostatic interpretation, suggesting that reduced physiological reserve in advanced age increases susceptibility to the same external workload.

Furthermore, these findings are consistent with Systems Gerontology, which views ageing as a dynamic interplay between behavioural routines, environmental exposures, and physiological responses (51). Our evidence demonstrates a multi-level interaction: the rural environment constrains behavioural options, behaviour shapes haemodynamic and metabolic stress, and these physiological pathways collectively elevate hypertension risk. In particular, the observed subgroup patterns—such as the more substantial labour effects in adults aged ≥70 years and stronger alcohol effects in men and leaner individuals—illustrate how intrinsic and extrinsic factors jointly regulate vulnerability across the ageing process.

Cross-cultural and genetic perspectives further contextualise these patterns. In contrast to the discretionary exercise common in high-income countries, rural Chinese activity is often necessity-driven, strenuous, and performed under climatic adversity, which helps explain the positive association between OPA and hypertension observed here. Similarly, East Asian–specific genetic factors, such as ALDH2 polymorphisms that impair alcohol metabolism, may amplify alcohol-related vascular responses (52, 53). While ALDH2 status could not be directly assessed in this dataset, these genetic pathways remain a plausible, hypothesis-generating mechanism for the observed associations.

Finally, the modest but independent associations of triglycerides and HDL-C with hypertension reinforce the concept that cardiovascular risk in ageing populations emerges from the interaction of behavioural strain, metabolic dysregulation, and environmental context. These patterns underscore the importance of place-based cardiovascular epidemiology, emphasising that behavioural risk cannot be disentangled from the environments in which behaviours occur. Together, this study highlights the need for contextually adaptive, environment-aware prevention strategies—such as workload modulation and targeted reduction of any weekly drinking—rather than universalised recommendations that may overlook the lived realities of rural older adults.

### 4.3 Mechanistic pathways linking behaviour, metabolism, and blood pressure

To interpret the associations between occupational (labour-type) physical activity (OPA), alcohol consumption, and hypertension observed in this rural geriatric cohort, we synthesised a dual-pathway conceptual framework grounded in established physiological literature (54). Given that the study did not include direct mechanistic biomarkers (e.g., neuroendocrine, inflammatory, or autonomic indicators), this framework should be viewed as a heuristic interpretation rather than a confirmed causal model.

#### 4.3.1 Behavioural–metabolic pathway

While moderate, voluntary, and recovery-supported physical activity is well documented to enhance lipid oxidation, HDL-mediated cholesterol transport, and metabolic efficiency, the activity captured in this cohort largely reflects obligatory, high-load agricultural labour (55, 56). Such OPA, often performed for protracted hours under cold-climate conditions, may plausibly diminish recovery capacity and impose cumulative metabolic strain; however, intermediate biomarkers (e.g., catecholamines, oxidative markers) were not measured.

Alcohol consumption displayed a modest metabolic signature partially consistent with recognised hepatic dysregulation pathways via the ADH–ALDH2 system (57, 58). The extended logistic models detailed in Section 3.8.2 help characterise the contributions of lipid biomarkers to hypertension risk within this cohort. Specifically, while triglycerides exhibited a modest but statistically independent association with hypertension, no such relationship was observed for LDL-C. Interestingly, HDL-C demonstrated an unexpected positive association with elevated blood pressure. This pattern is more plausibly attributable to external factors—such as antihypertensive medication exposure, reverse causation, or survivor bias—rather than a direct detrimental physiological effect of HDL-C itself. Such an interpretation is particularly relevant given the cross-sectional nature of the data and the inherent complexities of lipid metabolism in an older population.

#### 4.3.2 Haemodynamic and stress-load pathway

A more coherent explanatory pattern emerges from haemodynamic load and cumulative stress physiology. Repeated heavy labour—especially outdoors during Northeast China’s prolonged cold season—may plausibly elicit sustained sympathetic activation, cold-induced vasoconstrictive responses, elevated systemic vascular resistance, and baroreflex strain (59–61). These mechanisms align with a chronic stress-load model, in which non-volitional, environmentally imposed workload contributes to a steadily increasing haemodynamic burden.

The continuous blood pressure models (Supplementary Table S3) provide outcome-level support for this pathway by demonstrating that both SBP and DBP increase with higher OPA and drinking frequency, consistent with a cumulative pressor load rather than an isolated threshold phenomenon. Alcohol use may further potentiate these effects through autonomic imbalance, endothelial dysfunction, and acute fluctuations in vascular tone (62–64). The monotonic behavioural dose–response gradients and stable odds ratios observed across robustness checks (Sections 3.5–3.6) are congruent with a cumulative haemodynamic-stress pathway rather than a lipid-mediated mechanism. Although inflammatory and neurohumoral biomarkers were unavailable, the empirical patterns are broadly consistent with established conceptual models of cumulative physiological load in ageing populations.

#### 4.3.3 Sex-, age-, and BMI-specific vulnerability

The behavioural pathways described above were not uniform across subgroups (Section 3.7). Alcohol-related hypertension risk was concentrated in men, adults aged <70 years, and leaner individuals (BMI < 24 kg/m^2^). This pattern may reflect sex-based differences in drinking behaviours and sociocultural norms, and is potentially consistent with ALDH2-related metabolic sensitivity that is prevalent in East Asian populations (52, 65). In contrast, occupational physical activity (OPA) exhibited its strongest association with hypertension among adults aged ≥70 years, aligning with age-related declines in vascular compliance, autonomic buffering capacity, and overall physiological reserve that may heighten susceptibility to cumulative workload-related haemodynamic strain (66, 67).

Notably, associations for both exposures appeared attenuated among participants with a BMI ≥ 28 kg/m^2^. This observation may reflect treatment differences, survivorship dynamics, or other unmeasured factors, and should therefore be interpreted cautiously as hypothesis-generating rather than mechanistically definitive. Collectively, these findings reinforce the view that behavioural strain interacts with age-related vascular physiology and body composition to produce differential susceptibility across demographic groups. Consistent with this interpretation, Supplementary Figure S1 illustrates a steeper OPA–hypertension gradient at older ages, and Supplementary Table S5 provides statistical evidence that age modifies the physical activity association (OPA × age interaction).

#### 4.3.4 Integrated conceptual framework

Across these pathways, our findings support a coherent behavioural–physiological sequence in which environmental constraints shape daily behaviours, which in turn generate a cumulative haemodynamic load with at most modest metabolic strain; this cumulative burden was associated with elevated hypertension risk. This integrated interpretation is consistent with the approximately linear behavioural gradients observed for occupational physical activity (OPA) and alcohol frequency (Sections 3.2–3.4), the limited evidence that fasting lipid markers provide meaningful mediation of these associations (Section 3.8), and the stability of estimates across alternative hypertension definitions and sensitivity analyses (Section 3.6). It also aligns with the subgroup-specific vulnerability patterns by sex, age, and BMI (Section 3.7).

Taken together, the evidence supports a context-sensitive mechanistic narrative in which hypertension risk among rural older adults reflects the intersection of environmental constraints, non-volitional labour demands, habitual alcohol use, and age-related physiological vulnerability. This framework is intended as an interpretive, biologically plausible synthesis rather than a causal claim, and it provides the conceptual basis for the prevention implications discussed in Section 4.4 (5). Finally, the absence of a statistically significant OPA × alcohol interaction (Supplementary Table S4) suggests that prevention strategies should address both exposures as parallel and potentially cumulative targets, rather than presuming synergistic amplification on the multiplicative scale.

### 4.4 Implications, limitations, and future directions

#### 4.4.1 Public health and policy implications

The findings of this study challenge conventional assumptions regarding physical activity in ageing populations and highlight the need to distinguish between beneficial, recovery-supported exercise and compulsory, high-load labour. In this rural context, cardiovascular health may depend less on accumulating total activity volume than on achieving a “Healthy Labour Balance”—a concept emphasising appropriate intensity, sufficient recovery, environmental safety, and an age-appropriate workload (68). Consistent with the age-amplified OPA gradient (Supplementary Table S5; Supplementary Figure S1), workload-modulation interventions may be particularly relevant for the oldest adults, for whom incremental increases in OPA frequency correspond to steeper increases in predicted hypertension probability.

From a public health perspective, integrating routine alcohol-use screening, labour-intensity profiling (e.g., brief workload screening items), and bilateral blood pressure measurement (using the higher-arm value) into rural health surveillance could improve the identification of individuals at elevated vascular risk. Community-level prevention should prioritise structured, low-intensity activity programmes, evidence-based alcohol-reduction interventions, and cold-weather protection strategies, alongside strengthened access to primary and preventive care within township-level health systems. Because alcohol-related risk appears concentrated in specific subgroups—notably men, leaner individuals, and the younger older adults—targeted approaches (e.g., brief interventions and culturally adapted counselling) may be more efficient than uniform messaging.

These recommendations align with the WHO Healthy Ageing framework and the Healthy China 2030 agenda, while contributing to emerging international discussions on place-based cardiovascular prevention (68). Importantly, they emphasise that behavioural recommendations must be adapted to local labour patterns, climatic conditions, and social norms, rather than transferred wholesale from urban or high-income settings.

#### 4.4.2 Strengths and limitations

This study has several methodological strengths. It leveraged standardised, government-administered examination data collected under uniform protocols. Blood pressure was measured in both arms, and the higher-arm value was used for primary classification, consistent with AHA and BHS recommendations. Physiological and laboratory assessments were conducted under strict quality-control procedures, and behavioural exposures were assessed for both occupational physical activity (OPA) and alcohol use. The analytic strategy was rigorous, applying HC3 heteroscedasticity-consistent standard errors and confirming robustness across alternative hypertension definitions and multiple sensitivity analyses. In addition, subgroup analyses showed consistent heterogeneity patterns across sex, age, and BMI strata. Supplementary outcome operationalisations—including SBP-only/DBP-only definitions (Supplementary Table S2) and continuous blood pressure models (Supplementary Table S3)—further provide convergent evidence that the behavioural associations are not artefacts of a single diagnostic threshold.

Several limitations warrant cautious interpretation (69). The cross-sectional design precludes causal inference and limits temporal interpretation; therefore, residual confounding and reverse causation cannot be excluded. Behavioural exposures were self-reported, which may introduce recall and social-desirability bias. The anonymised secondary database did not include month- or season-specific examination timestamps; therefore, we could not formally evaluate seasonal variation (e.g., harvest periods or extreme winter cold) in occupational workload or OPA reporting, nor stratify analyses by agricultural or climatic period. Future surveillance with season-resolved timestamps or longitudinal follow-up would help disentangle climatic and agricultural-cycle influences on workload exposure. Moreover, key covariates—such as detailed dietary patterns, psychosocial stressors, medication adherence, and objective environmental exposures (e.g., cold exposure and workload intensity)—were not systematically measured. Accordingly, the proposed mechanistic pathways were inferred from epidemiological patterns rather than directly tested using biomarker assays (e.g., autonomic indicators or inflammatory cytokines). Finally, interaction models (Supplementary Tables S4, S5) assessed effect modification on the multiplicative scale; thus, the non-significant OPA × alcohol term should be interpreted as an absence of multiplicative synergy in these data rather than evidence against a potentially meaningful combined public-health burden, including additive joint effects. Collectively, these limitations highlight key empirical gaps and underscore the need for future multi-site longitudinal studies.

#### 4.4.3 Future directions

Future research should prioritise longitudinal cohort designs to characterise behavioural–metabolic–haemodynamic trajectories over time and to validate the dual-pathway framework proposed in this study. Integrating multi-omics approaches—including metabolomics, lipidomics, proteomics, and inflammatory profiling—may help clarify whether the observed behavioural associations operate predominantly through cumulative haemodynamic stress, metabolic dysregulation, or coupled pathways linking the two.

Objective exposure assessment should be strengthened using wearable sensors capable of capturing labour intensity, daily physical load, recovery duration, heart-rate variability, and cold-weather exposure in a low-burden manner suitable for rural settings (14). In parallel, AI-enabled modelling tools could support personalised risk stratification, provided they are implemented ethically and evaluated for calibration and clinical utility within local primary-care capacity constraints.

Given the pronounced sex- and age-specific vulnerability patterns, future intervention trials should test tailored strategies—for example, recovery-oriented activity programmes for older women and targeted alcohol-reduction interventions for high-risk subgroups such as men and the younger older adults. Incorporating community-based participatory approaches may further enhance feasibility, cultural acceptability, and adherence in resource-limited settings.

#### 4.4.4 Overall synthesis

Taken together, this study provides a behavioural–metabolic–environmental framework for understanding hypertension risk among rural older adults in Northeast China. Occupational physical activity (OPA) and alcohol consumption—behaviours shaped by persistent structural, environmental, and cultural constraints—appear to exert convergent haemodynamic and modest metabolic pressures that collectively elevate vascular risk (70).

These findings reinforce that hypertension prevention in rural settings cannot rely on generic recommendations (e.g., “increase physical activity” or “reduce drinking”) without accounting for the lived realities and structural exposures faced by older populations. Instead, effective strategies should be context-adapted, environmentally sensitive, and aligned with community labour patterns and social practices (70). Multiple supplementary analyses further supported the robustness of the observed associations. Specifically, findings were consistent under SBP-only/DBP-only definitions (Supplementary Table S2), and both OPA and alcohol frequency were linearly related to continuous SBP and DBP (Supplementary Table S3). The OPA–hypertension association strengthened with age (Supplementary Table S5; Supplementary Figure S1), whereas the absence of a multiplicative interaction (Supplementary Table S4) suggests that these exposures represent parallel, potentially cumulative targets for intervention.

By situating these behavioural gradients within a place-based framework, this study advances understanding of how cardiovascular risk emerges at the intersection of necessity-driven behaviour, environmental stressors, and ageing physiology, and it provides a foundation for future mechanistic research and policy innovation in rural geriatric health.

## 5 Conclusion

In this community-based study of rural older adults in Northeast China, occupational physical activity (OPA) and alcohol consumption were both positively and approximately linearly associated with hypertension. Each additional weekly episode of manual or agricultural labour, and each additional drinking occasion, was associated with higher odds of measured hypertension, with no evidence of a protective association at lower exposure levels within the observed range. Population-attributable fraction (PAF) estimates suggested that heavy, non-volitional labour could account for a substantial share of hypertension cases in this setting, with the burden further compounded by habitual weekly drinking.

These findings indicate that, in cold-climate agricultural communities, physical activity may function less as a health-promoting behaviour and more as a marker of cumulative workload and physiological strain. Prevention strategies should therefore prioritise the promotion of a “Healthy Labour Balance,” characterised by age-appropriate workload, adequate recovery, and reduced weekly alcohol use. Integrating bilateral blood pressure measurement, labour-intensity profiling, and routine alcohol-use screening into rural health services may improve early detection and risk stratification among older adults.

This study is limited by its cross-sectional design, reliance on self-reported behaviours, and the absence of mechanistic biomarkers. Future longitudinal studies and intervention trials are needed to clarify causal pathways linking OPA, alcohol use, and vascular ageing. Overall, these results support the need for context-specific, environment-aware hypertension prevention strategies in resource-constrained rural communities.

## Data Availability

The data analysed in this study is subject to the following licences/restrictions: the dataset consists of de-identified health examination records obtained from the Dongcheng Community Health Service Center (Wangkui County, Heilongjiang Province, China). Under Chinese data protection regulations and institutional restrictions, these data cannot be publicly shared or transferred to third parties. Access to the dataset requires formal written authorization from the health service center, and therefore the dataset is not available for public release. Due to legal and institutional restrictions, the dataset cannot be accessed by external researchers; therefore, no contact person, email address, or website for data access is available.
