# Correction: Labour-type physical activity, alcohol use and hypertension in rural older adults in Northeast China

**DOI:** 10.3389/fpubh.2026.1821356

**Published:** 2026-03-18

**Authors:** Yongheng Zhao, Gaixia Hou, Yimeng Gu, Zhuangzhuang Guo, Dehui Zhang, Xuefeng Xi, Limeng Liu, Lizhen Ning

**Affiliations:** 1 School of Wushu, Henan University, Kaifeng, China; 2 Institute of Gerontology, Henan University, Kaifeng, China; 3 Graduate School, Kyungil University, Gyeongsan-si, Republic of Korea; 4 Dongcheng Community Health Service Center, Wangkui County, Heilongjiang, China; 5 School of Physical Education and Health Sciences, Mudanjiang Normal University, Mudanjiang, China; 6 School of Physical Education, Suihua University, Suihua, China

**Keywords:** alcohol consumption, cold-climate environment, hypertension, occupational physical activity, population-attributable risk, rural older adults

In the published article, there was a mistake in the ethics approval information reported in the **Methods** section and the **Ethics statement**. The ethics approving body and approval number were inconsistently reported.

In **Methods**, *2.5 Ethical approval and data governance*, the ethics approval sentence was erroneously given as:

“The secondary analysis protocol was reviewed and approved by the Ethics Committee of Henan University (HU-REC-2025-011).”

The correct ethics approval sentence is:

“The secondary analysis protocol was reviewed and approved by the Henan University Biomedical Research Ethics Sub-Committee (approval no. HUSOM2025-929).”

In the **Ethics statement**, the ethics statement was erroneously given as:

“The studies involving humans were approved by the Dongcheng Community Health Service Center, Wangkui County, Heilongjiang Province, China (approval no. HUSOM2025-929). The studies were conducted in accordance with the local legislation and institutional requirements. Written informed consent for participation was not required from the participants or the participants' legal guardians/next of kin in accordance with the national legislation and institutional requirements.”

The correct **Ethics statement** is:

“The studies involving humans were approved by the Henan University Biomedical Research Ethics Sub-Committee (approval no. HUSOM2025-929). The studies were conducted in accordance with the local legislation and institutional requirements. Written informed consent for participation was not required from the participants or the participants' legal guardians/next of kin in accordance with the national legislation and institutional requirements.”

The original version of this article has been updated.

