# Supplementary material for: Labour-type physical activity, alcohol use and hypertension in rural older adults in Northeast China
Source: Front Public Health. 2026 Feb 25;14:1748721. doi: 10.3389/fpubh.2026.1748721 (PMC12975895; doi:10.3389/fpubh.2026.1748721)
Supplement: Supplementary file 1 [file Supplementary_file_1.docx]

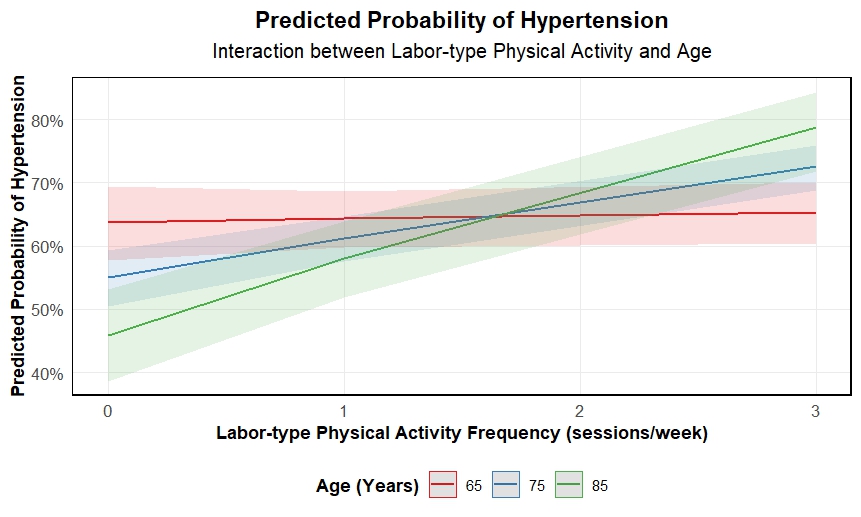


**Figure S1. Predicted probability of hypertension across labor-type physical activity frequency by age (proxy workload-heterogeneity interaction).** This figure visualizes the interaction between weekly labor-type physical activity (PA) frequency and age in relation to measurement-defined hypertension, based on an HC3-robust multivariable logistic regression model including the term PA × age. Curves depict model-based predicted probabilities of hypertension across the observed range of PA frequency at representative ages (e.g., 65, 75, and 85 years), with other covariates held constant at typical values. The positive PA × age interaction indicates that the association between more frequent labor-type PA and hypertension becomes stronger at older ages, consistent with an age-amplified workload–vascular risk pattern in this rural cohort. Models were adjusted for sex, BMI, hemoglobin (Hb_gL), and winsorized resting heart rate (HR_winsor); standard errors were estimated using HC3 heteroscedasticity-consistent covariance.
