# Supplementary material for: Labour-type physical activity, alcohol use and hypertension in rural older adults in Northeast China
Source: Front Public Health. 2026 Feb 25;14:1748721. doi: 10.3389/fpubh.2026.1748721 (PMC12975895; doi:10.3389/fpubh.2026.1748721)
Supplement: Supplementary file 2 [file Table_1.docx]

**Table S1. Variable mapping, harmonisation, and dataset integration for reproducible analysis.**

| **Variable domain** | **Raw variable(s)** | **Standardised variable (used in analysis)** | **Source file/table** | **Processing / harmonisation rule** | **Primary use in manuscript** |
| --- | --- | --- | --- | --- | --- |
| Identifiers | pid / id_number | id | Lifestyle + Physical exam | Unique key for merging datasets | All analyses |
| Demographics | sex / gender | sex (Men/Women) | Physical exam | Re-coded and validated for consistency (Men/Women) | Table 1; Section 3.1 |
|  | age | age (years) | Lifestyle | Continuous; analytic subset restricted to ≥65 years | Methods; Sections 3.1–3.3 |
| Anthropometrics | height, weight | bmi (kg/m²) | Lifestyle | BMI = weight / height² (kg/m²) | Main adjusted models |
|  | waist | waist_circumference (cm) | Lifestyle | As measured | Supplementary exploration |
| Blood pressure | sbp_left, sbp_right, dbp_left, dbp_right | SBP_higharm, DBP_higharm | Physical exam | Higher-arm value used for primary definition; mean-arm used in sensitivity analyses | Table 1; Sections 3.1–3.7 |
| Heart rate | pulse_rate | HR_raw → HR_winsor | Physical exam | Numeric conversion; winsorised at 1st–99th percentiles | Table 1; adjusted models |
| Haematology | hemoglobin | Hb_gL | Physical exam | Unit harmonisation to g/L where required | Table 1; adjusted models |
| Lipids & glucose | tg, tc, hdl, ldl, fpg, rbg | TG, TC, HDL_C, LDL_C, FBG, RBG | Physical exam | Unit check (mmol/L); kept on original scale in main models | Section 3.8; supplementary |
| Inflammation / liver | hs_crp, alt, ua | hs_crp, alt, ua | Physical exam | Natural log transformation if markedly skewed | Supplementary analyses |
| Lifestyle | pa_level, smoking, drinking | exercise_freq, drink_freq, smoking | Lifestyle | Re-coded to match analysis scale; exposures treated continuous in main models | Sections 3.2–3.4 |
| Disease diagnosis | hypertension, dyslipidemia, diabetes, cvd_risk2plus | binary indicators (0/1) | Lifestyle + Medical record | Harmonised coding; descriptive only unless specified | Section 3.1 summary |
| Medication use | antihypertensive, lipid_drug | binary (0/1) | Medical record | Used for sensitivity/subsample analyses | Sections 3.6, 3.8 |
| Merge rule | — | — | — | Merged by **id** and exam year; restricted to participants with ≥1 valid BP record; analytic samples vary by variable completeness | Overall pipeline |

**Note：** Variables unavailable in the physical examination dataset (e.g., age, exercise/drinking frequency, smoking, BMI inputs) were sourced from the lifestyle questionnaire. Missingness for core variables used in the primary pipeline (blood pressure, physical activity, alcohol use, age, sex, BMI, haemoglobin, resting heart rate) was <5%; no imputation was performed. This mapping enables full reproducibility of the descriptive statistics (Table 1) and the primary regression models (Tables 2–4; Figures 2–3).

**Table S2. Multivariable logistic regression of labor-type physical activity and alcohol drinking frequency with SBP-only and DBP-only hypertension (HC3-robust SE).**

| **Outcome** | **Exposure (per +1 unit/week)** | **OR (95% CI)** | **β (log-odds)** | **p-value** |
| --- | --- | --- | --- | --- |
| SBP-only HTN (SBP ≥140 mmHg) | Labor-type PA frequency | 1.19 (1.12, 1.27) | 0.1767 | <0.001 |
|  | Alcohol drinking frequency | 1.16 (1.01, 1.33) | 0.1463 | 0.035 |
| DBP-only HTN (DBP ≥90 mmHg) | Labor-type PA frequency | 1.16 (1.08, 1.24) | 0.1476 | <0.001 |
|  | Alcohol drinking frequency | 1.15 (1.02, 1.31) | 0.1421 | 0.025 |

**Note：** Values are adjusted odds ratios (ORs) with 95% confidence intervals (95% CI). SBP-only hypertension was defined as SBP ≥140 mmHg irrespective of DBP; DBP-only hypertension was defined as DBP ≥90 mmHg irrespective of SBP, both based on higher-arm blood pressure. Models were estimated using multivariable logistic regression with HC3 heteroscedasticity-consistent standard errors. All models were adjusted for age (years), sex, BMI (kg/m²), haemoglobin (g/L), and winsorised resting heart rate (1st–99th percentile). Physical activity and alcohol exposures were modelled as continuous variables (per +1 unit/week). Complete-case analysis was applied (N ≈ 2,19×). Two-sided p-values were used; p < 0.05 was considered statistically significant.

**Table S3. Continuous Blood Pressure Models (HC3-robust linear regression)**

**Table S3A. Outcome: Systolic blood pressure (SBP, mmHg; higher arm)**

| **Predictor** | **β (95% CI)** | **p-value** |
| --- | --- | --- |
| Labor-type PA frequency (per +1 session/week) | **1.49 (0.87, 2.11)** | <0.001 |
| Alcohol drinking frequency (per +1 time/week) | **1.73 (0.49, 2.97)** | 0.006 |
| Age (years) | 0.21 (0.06, 0.36) | 0.006 |
| Sex (Men vs Women)* | -1.95 (-3.83, -0.07) | 0.042 |
| BMI (kg/m²) | 0.81 (0.54, 1.08) | <0.001 |
| Haemoglobin (g/L) | 0.10 (0.03, 0.17) | 0.005 |
| Resting HR (winsorised) | 0.07 (-0.01, 0.15) | 0.102 |

**Table S3B. Outcome: Diastolic blood pressure (DBP, mmHg; higher arm)**

| **Predictor** | **β (95% CI)** | **p-value** |
| --- | --- | --- |
| Labor-type PA frequency (per +1 session/week) | **0.79 (0.46, 1.12)** | <0.001 |
| Alcohol drinking frequency (per +1 time/week) | **0.88 (0.23, 1.53)** | 0.008 |
| Age (years) | -0.05 (-0.14, 0.04) | 0.249 |
| Sex (Men vs Women)* | 0.50 (-0.55, 1.55) | 0.353 |
| BMI (kg/m²) | 0.46 (0.31, 0.60) | <0.001 |
| Haemoglobin (g/L) | 0.14 (0.10, 0.18) | <0.001 |
| Resting HR (winsorised) | 0.12 (0.08, 0.17) | <0.001 |

**Note：**Values are unstandardised regression coefficients β (mmHg) with 95% confidence intervals from ordinary least squares linear regression with HC3 robust standard errors. Outcomes were continuous higher-arm SBP/DBP. Models adjusted for age, sex, BMI, haemoglobin, and winsorised resting heart rate (1st–99th percentile). Physical activity and alcohol frequency were modelled as continuous variables (per +1 unit/week). Complete-case analysis was applied (N ≈ 2,19×). *Coding for sex should match the main dataset (e.g., Men as reference); keep consistent with your regression coding.

**Table S4. Multiplicative interaction between labor-type physical activity and alcohol drinking frequency on hypertension (HC3-robust logistic regression).**

| **Term** | **OR (95% CI)** | **β (log-odds)** | **p-value** |
| --- | --- | --- | --- |
| Labor-type PA frequency | 1.24 (1.16, 1.32) | 0.2129 | <0.001 |
| Alcohol drinking frequency | 1.42 (1.04, 1.92) | 0.3475 | 0.025 |
| PA × Alcohol | 0.93 (0.83, 1.04) | -0.0752 | 0.194 |
| Age (years) | 0.99 (0.98, 1.01) | -0.0066 | 0.410 |
| Sex (Men vs Women)* | 0.82 (0.66, 1.00) | -0.2043 | 0.053 |
| BMI (kg/m²) | 1.08 (1.05, 1.11) | 0.0749 | <0.001 |
| Haemoglobin (g/L) | 1.02 (1.01, 1.03) | 0.0194 | <0.001 |
| Resting HR (winsorised) | 1.01 (1.00, 1.01) | 0.0053 | 0.206 |

**Note：** Values are adjusted odds ratios (ORs) with 95% confidence intervals from multivariable logistic regression with HC3 robust standard errors. Hypertension was defined as higher-arm SBP ≥140 mmHg and/or DBP ≥90 mmHg. The multiplicative interaction term (PA × Alcohol) tests whether the association between labor-type physical activity frequency and hypertension differs by alcohol drinking frequency. Models adjusted for age, sex, BMI, haemoglobin, and winsorised resting heart rate. Physical activity and alcohol frequency were modelled as continuous variables (per +1 unit/week). Complete-case analysis was applied (N ≈ 2,19×). *Sex coding should be consistent with the primary model.

**Table S5. Interaction between labor-type physical activity frequency and age as a proxy test of workload-duration heterogeneity (HC3-robust logistic regression).**

| **Term** | **OR (95% CI)** | **β (log-odds)** | **p-value** |
| --- | --- | --- | --- |
| Labor-type PA frequency | 0.22 (0.10, 0.50) | -1.4983 | <0.001 |
| Age (years) | 0.96 (0.94, 0.98) | -0.0367 | <0.001 |
| Alcohol drinking frequency | 1.20 (1.03, 1.39) | 0.1828 | 0.016 |
| PA × Age | 1.02 (1.01, 1.04) | 0.0234 | <0.001 |
| Sex (Men vs Women)* | 0.82 (0.66, 1.00) | -0.2044 | 0.054 |
| BMI (kg/m²) | 1.07 (1.04, 1.11) | 0.0713 | <0.001 |
| Haemoglobin (g/L) | 1.02 (1.01, 1.03) | 0.0189 | <0.001 |
| Resting HR (winsorised) | 1.00 (1.00, 1.01) | 0.0046 | 0.277 |

**Note：** Values are adjusted odds ratios (ORs) with 95% confidence intervals from multivariable logistic regression with HC3 robust standard errors. The interaction term (PA × Age) evaluates whether the association between labor-type physical activity frequency and hypertension varies by age, serving as a proxy indicator of workload-duration susceptibility in this rural cohort. Models adjusted for age, sex, BMI, haemoglobin, and winsorised resting heart rate. Physical activity and alcohol frequency were modelled as continuous variables (per +1 unit/week). Predicted probabilities derived from this model (e.g., age = 65/75/85 years) are presented in Figure S1. Complete-case analysis was applied (N ≈ 2,19×). *Sex coding should be consistent with the primary model. In models with continuous interaction terms, the main effect of PA represents the effect at age=0; interpretation should rely on marginal effects/predicted probabilities across observed age values (Figure S1)
